# Infrared photons generated by voltage-gated sodium channel dynamics: a hypothesis for cooperative electrodynamic interactions at the axon initial segment

**DOI:** 10.3389/fnhum.2026.1772352

**Published:** 2026-09-24

**Authors:** Daya Shankar Gupta

**Affiliations:** School of Pharmacy, South University, Savannah, GA, United States

**Keywords:** binding, consciousness, Dicke radiation, neural synchrony, photon entanglement, superradiance, temporal processing of information, voltage-gated sodium channels

## Abstract

Voltage-gated sodium (NaV) channels are essential for neuronal excitability and action-potential generation, yet the electrodynamic consequences of voltage-sensor motion remain largely unexplored. Here, we propose a biophysically grounded hypothesis in which transient electromechanical dynamics of NaV-channel voltage sensors give rise to weak cooperative electrodynamic interactions at the axon initial segment (AIS). Specifically, we consider the behavior of conserved arginine gating charges within the S4 voltage-sensor segment as they traverse the hydrophobic constriction site (HCS) and charge-transfer center (CTC) during membrane depolarization. Under subthreshold conditions, local electrostatic and *π*–cation interactions may transiently stabilize intermediate states of S4 motion, permitting brief reversible excursions (“micro-reversals”) of gating charges within the confined high-field environment of the HCS. Based on known structural properties of the voltage-sensing domain and experimentally characterized vibrational modes of the arginine guanidinium group, we propose that these micro-reversal dynamics may generate transient oscillating dipoles capable of weak electromagnetic emission in the mid-infrared range. Because NaV channels are densely organized within the AIS and occupy a subwavelength volume relative to the predicted emission wavelength, subsets of emitters may experience similar local electric fields, orientations, and transition frequencies. Under these conditions, transient phase alignment and weak field-mediated coupling among neighboring emitters could, in principle, produce extremely weak time-locked mid-infrared emission and, under conditions supporting transient cooperative emission, potentially exhibit nonclassical photon statistics. These proposed signatures are hypothetical predictions of the present framework and require experimental verification. The proposed mechanism does not require long-lived quantum coherence, sustained entanglement, or quantum computation. Rather, it provides a testable framework that links established NaV-channel biophysics with experimentally accessible electromagnetic observables. We present order-of-magnitude estimates of charge acceleration, radiated energy, and potential cooperative enhancement and outline experimentally testable predictions, including time-locked mid-infrared emission, nonclassical photon statistics, dependence on AIS organization, and sensitivity to pharmacological or genetic perturbation of NaV-channel function. Collectively, these predictions provide a basis for evaluating whether transient cooperative electrodynamic interactions occur during neuronal activity and, if so, whether they have any functional relevance for neural information processing, including feature integration, perceptual awareness, or voluntary motor control.

## 1 Introduction

Perceptual experience, or *phenomenal content*, refers to the subjective, first-person qualities of sensation—what it is like to see a color, hear a sound, or feel a texture. In contemporary neuroscience, such experiences are generally associated with distributed and coordinated activity across cortical networks, particularly within thalamocortical and corticocortical circuits. A substantial body of work links perceptual states to structured population activity, recurrent processing, and large-scale synchronization, especially in the gamma and beta frequency ranges (Singer, 1999; Fries, 2005; Dehaene and Changeux, 2011; Koch et al., 2016; Haggard, 2017; Gupta, 2022; Gupta et al., 2023). Related frameworks, including communication-through-coherence, further suggest that synchronization enhances effective information transfer among distributed brain regions by aligning neuronal excitability in time (Fries, 2005, 2015).

Despite extensive proposals involving neural synchrony, recurrent processing, and large-scale network dynamics, no current framework provides a fully specified mechanistic account of how distributed neural activity is coordinated to produce unified perceptual experience (Treisman, 1996; Singer, 1999; Fries, 2005; Tononi, 2008; Dehaene and Changeux, 2011).

Against this backdrop, several theoretical approaches have explored whether additional physical mechanisms beyond classical electrophysiology might transiently enhance neural coordination. Importantly, these frameworks do not propose that the brain operates as a quantum computer in the conventional computational sense. Rather, they examine whether specific biological structures may support ultrafast, spatially constrained quantum or near-quantum correlations that could, in principle, influence perceptual integration and conscious awareness (Freitas da Rocha et al., 2001; Atmanspacher, 2020; Fields et al., 2022; Liu et al., 2024; Neven et al., 2024). Another theoretical study has argued that quantum-like, rather than purely classical, computational frameworks may help explain the remarkable energy efficiency of neural information processing (Stoll, 2024a). According to this proposal, the computational capabilities of the brain appear disproportionately high relative to its energy consumption, motivating consideration of quantum-inspired mechanisms as a potential contributor to neural efficiency.

Motivated by these observations, we propose a testable, biophysically grounded framework in which transient electromechanical dynamics of voltage-gated sodium (NaV) channels may contribute to rapid neural coordination at the axon initial segment (AIS), where synaptic inputs are integrated (Kole et al., 2008; Kole and Stuart, 2012). In this model, classical mechanisms such as oscillatory synchrony continue to mediate information transfer and communication across neuronal circuits (Fries, 2005, 2015), while ultrafast electrodynamic processes are hypothesized to arise from the unique voltage-sensor dynamics of NaV channels (Table 1). These transient electrodynamic processes are proposed as a potential physical correlate of neuronal information processing at the AIS; however, any relationship to higher-order neural functions, including conscious awareness, remains entirely speculative and requires substantial experimental investigation.

**Table 1:** Distinctive features of S4 gating charges that motivate the proposed electrodynamic hypothesis.

Feature	Relevance to the proposed framework
Voltage-dependent movement	Conserved S4 arginine residues undergo coupled translational and rotational movements in response to changes in membrane potential, unlike arginine residues in most proteins.
Traversal of the HCS/CTC	Gating charges pass through the narrow HCS containing the conserved charge-transfer center, creating a highly localized electromechanical environment.
Intense local electric field	Most of the transmembrane voltage drops across the HCS, exposing gating charges to electric fields on the order of 10^7^–10^8^ V/m that may enhance polarization through the vibrational Stark effect.
Metastable intermediate states	Experimental and structural studies indicate that S4 activation proceeds through intermediate conformations, providing a potential basis for transient dwell times during subthreshold activation.
Reversible subthreshold dynamics	Gating-current measurements demonstrate that voltage-sensor movements can be reversible before channel opening, providing the experimental basis for the proposed micro-reversal hypothesis.
High density at the AIS	Approximately 10^4^–10^5^ NaV channels are concentrated at the AIS, creating a sufficiently large ensemble for possible collective electrodynamic effects.
Ordered nanoscale organization	Ankyrin-G and the actin–spectrin cytoskeleton organize NaV channels into a quasi-periodic arrangement that may facilitate transient coupling among neighboring voltage sensors.
Shared membrane-voltage drive	Neighboring voltage sensors experience nearly identical membrane-potential fluctuations during synchronized synaptic input, increasing the likelihood of similar transition frequencies and transient phase alignment.
Unique combination of properties	No other known arginine-containing protein combines voltage-dependent motion, confinement within a high-field HCS, reversible gating dynamics, dense nanoscale organization, and synchronized activation, making S4 gating charges a uniquely suitable candidate for the proposed mechanism.

Specifically, conserved arginine gating charges within the voltage-sensing domain traverse a highly confined hydrophobic constriction site containing intense local electric fields and a conserved charge-transfer center (CTC) (Bezanilla, 2000; Catterall, 2012; Yarov-Yarovoy et al., 2012; Catterall et al., 2020). Under subthreshold depolarization, these gating charges may undergo brief reversible transitions over sub-nanometer distances, producing transient acceleration of their associated charge distributions within the geometrically constrained environment of the voltage sensor.

Importantly, the present framework does not invoke long-lived quantum coherence or sustained quantum computation. Instead, it proposes that transient cooperative electrodynamic interactions among densely packed NaV channels at the AIS could briefly emerge during synaptic integration under highly constrained conditions (Table 2). Because the AIS contains approximately $10^{4}–10^{5}$ NaV channels arranged in a dense lattice-like organization, weak electromagnetic coupling among neighboring voltage sensors may be physically plausible over short spatial and temporal scales. If such cooperative interactions occur, they are predicted to produce only extremely weak physical signatures, including time-locked mid-infrared emission and, potentially, nonclassical photon statistics associated with voltage-sensor activation. These predictions remain hypothetical and are presented as experimentally testable consequences of the proposed framework rather than established properties of NaV-channel function. By linking established NaV-channel biophysics to specific experimental predictions, the present framework provides a basis for investigating whether transient electromechanical dynamics at the AIS contribute to neural information processing across molecular and cellular scales.

**Table 2:** Proposed conditions favoring transient cooperative electrodynamic interactions among neighboring NaV-channel emitters at the AIS.

Condition	Rationale within the proposed framework
Near-synchronous activation of neighboring NaV channels	Shared subthreshold membrane depolarization at the AIS may activate nearby voltage sensors within the same sub-picosecond coherence window.
Subthreshold membrane potential	Near-threshold depolarization increases the probability of transient metastable S4 states and proposed micro-reversal dynamics before full channel activation.
Similar transition frequencies	Neighboring S4 emitters exposed to similar local electric fields are expected to exhibit comparable vibrational transition frequencies, a prerequisite for cooperative emission.
Similar dipole orientations	The common orientation of voltage sensors within the membrane and the local electric field may partially align transition dipoles, increasing constructive interference.
Common electromagnetic environment	The confined HCS and the shared membrane environment provide a common local electromagnetic field that may facilitate weak coupling.
Subwavelength spatial organization	The AIS diameter (~0.5–1 μm) is substantially smaller than the predicted mid-infrared wavelength (~3–10 μm), allowing neighboring emitters to couple to nearly the same electromagnetic phase.
High local NaV-channel density	Dense clustering (approximately 10^4^–10^5^ channels per AIS increases the probability that subsets of emitters satisfy the required spatial and temporal conditions).
Reduced structural heterogeneity within local subdomains	Local groups of channels sharing similar membrane orientation and cytoskeletal organization are more likely to possess similar electrodynamic properties than the entire AIS ensemble.
Transient coherence interval	Cooperative interactions, if present, are expected to persist only during brief (~0.1–1 ps) coherence windows before environmental decoherence dominates.
Rapid environmental decoherence	Thermal fluctuations, protein vibrations, electrostatic noise, and residual hydration are expected to terminate cooperative dynamics rapidly, restricting any correlations to short temporal intervals.

## 2 Voltage-gated sodium channels act as an integrative substrate

The present framework proposes that voltage-gated sodium (NaV) channels, particularly their voltage-sensing domains (VSDs), provide a specialized biophysical substrate capable of integrating synaptic activity through transient electromechanical dynamics. To establish the basis for this hypothesis, the following sections review the structural organization and functional properties of the VSD that are relevant to S4 gating-charge movement and the proposed mechanism.

### 2.1 Structure of the voltage sensor domain

NaV channels are composed of a pore-forming *α*-subunit organized into four homologous domains, each containing six transmembrane segments (S1–S6) (Numa and Noda, 1986) (Figure 1). The S1–S4 segments form the voltage-sensing domain (VSD), whereas S5–S6 constitute the ion-conducting pore (Catterall, 2000). Within the VSD, the S4 segment contains multiple conserved arginine residues that serve as gating charges, responding to changes in membrane potential through coupled translational and rotational movements (Bezanilla, 2000). During activation, S4 undergoes an outward displacement of approximately 10–12 Å across the membrane electric field, accompanied by helical rotation and a partial conformational transition from a 3₁₀ helix to an *α*-helix near its extracellular end (Schwaiger et al., 2011).

**Figure 1:**
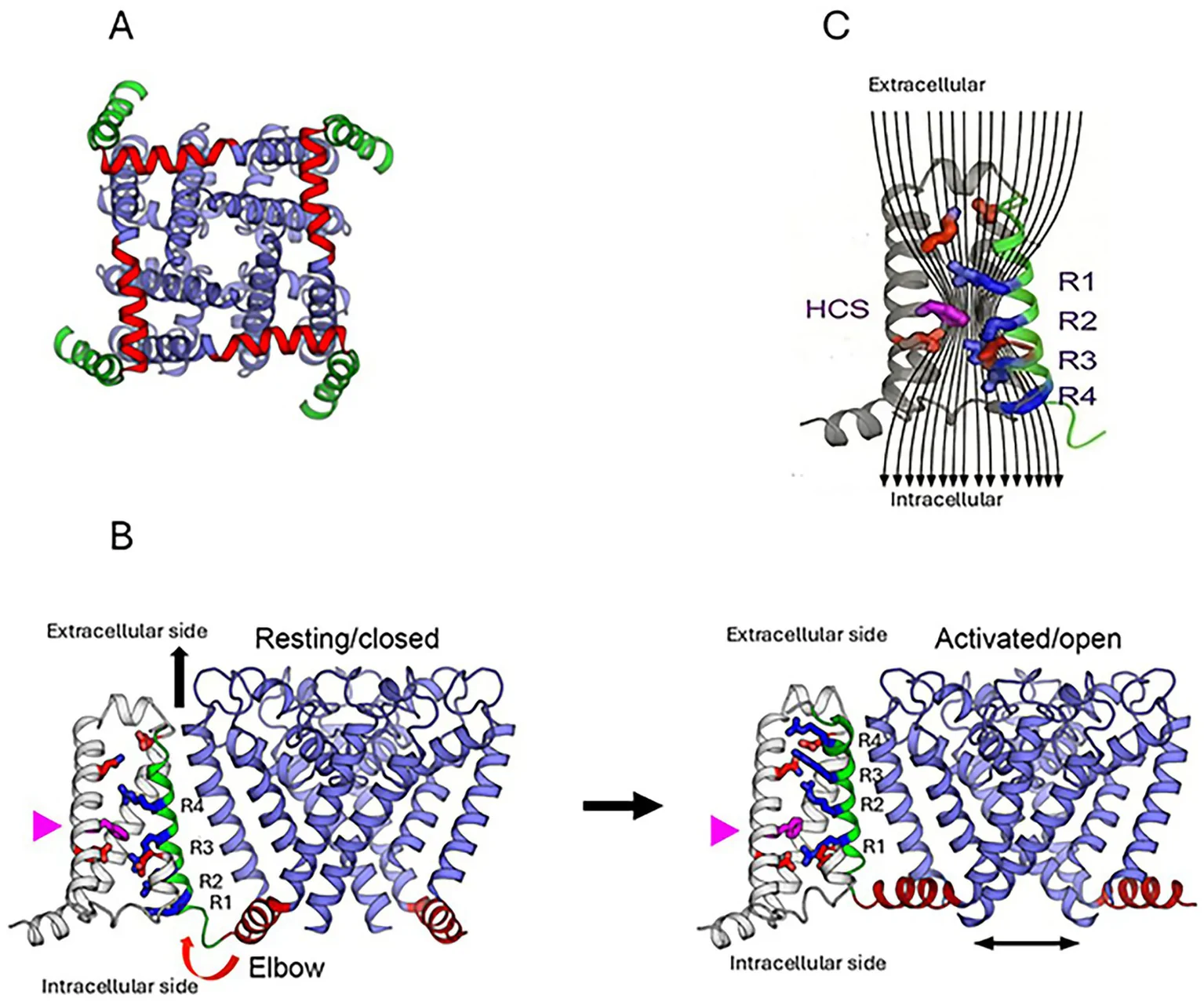
Voltage-sensor dynamics and local electric field at the hydrophobic constriction site (HCS). **(A)** Cross-sectional view of a vertebrate voltage-gated sodium channel. NaV channels are composed of a pore-forming *α*-subunit organized into four homologous domains (DI – IV), each containing six transmembrane segments (S1–S6). S4 segment, which contains gating charges in shown in green. Accordingly, there are four emitters on each NaV channel. **(B)** Schematic representation of the sliding-helix mechanism of voltage sensing. Upon membrane depolarization, the inward electrostatic force acting on the positively charged S4 gating charges—arginine residues (R1–R4), each containing a guanidinium side chain—is reduced. This change permits outward translation and rotation of the S4 helix, which carries the gating charges across the membrane electric field. During this process, the gating charges traverse the hydrophobic constriction site (HCS), a narrow region formed by a cluster of conserved hydrophobic residues from multiple transmembrane segments. A key phenylalanine residue (magenta color arrow) within this region constitutes the charge-transfer center (CTC) and provides a primary interaction site for arginine gating charges via electrostatic and *π*–cation interactions. The passage of gating charges through the CTC represents a significant energetic barrier, leading to transient slowing of S4 movement and increased dwell time in intermediate states. Within this metastable regime, small-amplitude, ultrafast fluctuations (“micro-oscillations”) of the gating charges may occur. **(C)** Illustration of the highly concentrated electric field within the HCS. Due to the steep voltage drop across this nanometer-scale region, the local electric field reaches intensities on the order of 10^7^–10^8^ V/m. This strong field enhances polarization of the positively charged guanidinium groups of arginine side chains and influences their orientation. During transient fluctuations near the CTC, these side chains may undergo rapid reorientation along local electric field lines, resulting in oscillatory charge motion. Such dynamics may, in principle, contribute to enhanced electromechanical coupling and transient electromagnetic emission. Panels **(A)**, **(B)** and **(C)** are adapted from the conformational cycle model reported by Catterall et al. (2020).

As illustrated in Figure 1, the VSD contains a highly conserved phenylalanine charge-transfer center (CTC), structurally associated with the hydrophobic constriction site (HCS), which together define the narrowest region along the gating charge pathway. This region is formed by a cluster of conserved hydrophobic residues, including a critical phenylalanine in the S2 segment, and functions as a low-dielectric barrier separating intracellular and extracellular aqueous vestibules (Catterall, 2012; Yarov-Yarovoy et al., 2012). The physical dimensions of the HCS are on the order of a few ångströms, with an estimated axial thickness of ~3–5 Å and a comparable lateral diameter, consistent with hydrophobic gating regions in ion channels (Beckstein and Sansom, 2006).

Such sub-nanometer confinement concentrates a substantial fraction of the transmembrane potential (~70–100 mV) across this short distance, generating a highly localized electric field near the CTC-phenylalanine region (Armstrong et al., 2016). This interpretation is consistent with structural and computational evidence indicating that S4 gating charges traverse the constriction through sequential ion-pair exchanges over angstrom-scale steps, implying that the principal voltage drop occurs within this narrow region (Yarov-Yarovoy et al., 2012). Consequently, the CTC may be viewed as a nanoscale electrostatic bottleneck in which geometric confinement and reduced dielectric screening compress electric field lines, generating one of the most intense localized electric field environments in biological systems.

### 2.2 S4 movement, subthreshold dynamics, and reversal

Membrane depolarization drives outward translational and rotational movement of the S4 voltage-sensor segment. While full activation leads to pore opening and action-potential initiation, experimental studies have demonstrated that subthreshold depolarizations can produce gating-charge movement without channel opening (Armstrong and Bezanilla, 1973, 1974; Delemotte et al., 2011; Bezanilla, 2018; Catterall et al., 2020; Heiss and Flucher, 2025). More recent structural studies further indicate that gating currents arise from voltage-dependent movement of S4 gating charges through multiple intermediate conformational states within the voltage-sensing domain of voltage-gated sodium channel (Biswas et al., 2026).

The S4 reversals are predicted to occur most readily at the AIS, where synchronous synaptic inputs are integrated to reach threshold to determine action potential initiation. Near the threshold, fluctuating membrane potentials may repeatedly bias the S4 segment between forward progression and transient reversal, thereby increasing the probability of localized oscillatory dynamics at CTC (Figure 2). In contrast, during axonal propagation following spike initiation, membrane depolarization is rapid and largely monotonic, driving S4 segments outward toward their activated state without substantial reversal behavior.

**Figure 2:**
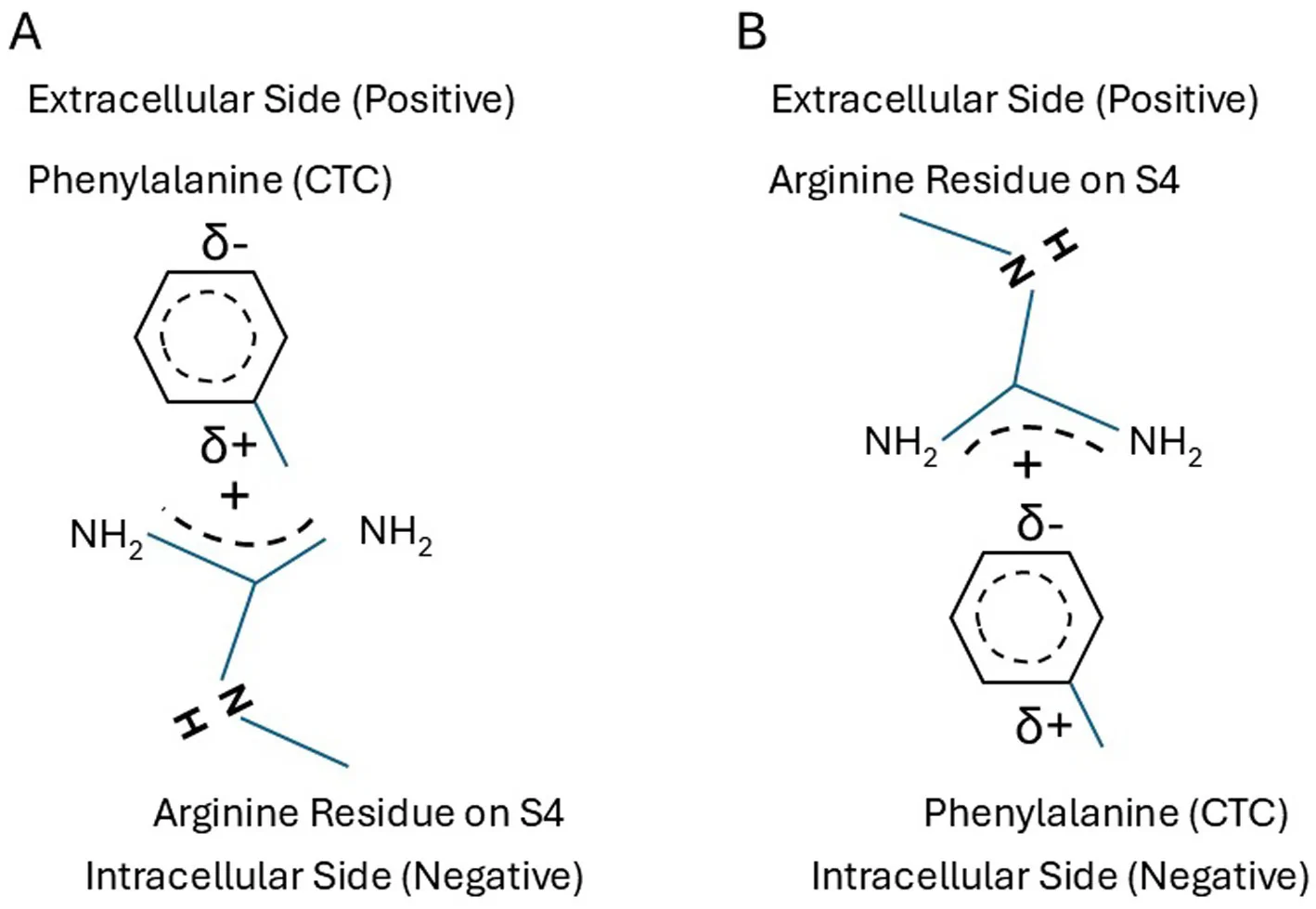
Figure illustrates the proposed interactions between the positively charged arginine gating residues of the S4 segment and the conserved phenylalanine residue within the charge-transfer center (CTC) during voltage-sensor activation. Within the highly confined, high-electric-field environment of the hydrophobic constriction site (HCS), the local electrostatic environment is expected to influence both the guanidinium charge distribution of arginine and the electronic properties of the phenylalanine aromatic ring. In the present framework, the intense electric field may transiently polarize the aromatic *π*-electron system, although the magnitude and functional significance of this effect have not been experimentally established. In **(A)**, the arginine gating charge approaches the conserved phenylalanine while traversing the narrow HCS. The combined effects of the local electric field, transient interactions with acidic countercharges, steric confinement, and the hydrophobic lock formed by the phenylalanine residue are proposed to generate a localized energetic barrier. Within the present framework, these interactions may transiently slow outward S4 movement, allowing the gating charge to occupy a metastable intermediate configuration. Under subthreshold depolarization, this metastable state is hypothesized to permit localized sub-ångström reversible excursions (“micro-reversals”) of the gating charge before continued outward translocation. In **(B)**, continued outward movement of the gating charge occurs following transient rotation (“flipping”) of the phenylalanine aromatic ring, consistent with the steered molecular dynamics simulations of Schwaiger et al. (2011). After the arginine side chain traverses the CTC, transient electrostatic interactions between the guanidinium group, nearby countercharges, and the aromatic residue may further stabilize the gating charge before the voltage sensor continues its activation pathway. Although direct *π*–cation interactions between the conserved phenylalanine and the arginine guanidinium group have not been demonstrated experimentally in voltage-gated ion channels, such interactions remain chemically plausible and are included here as a hypothetical component of the proposed mechanism.

## 3 Photon emission from oscillating arginine side chains

### 3.1 Interaction between arginine side chains with the CTC during S4 movement

Structural and functional studies indicate that the CTC constitutes a critical energetic checkpoint encountered by S4 gating charges during voltage-sensor activation (Tao et al., 2010) (Figure 2). Rather than occurring as a smooth continuous displacement, S4 activation proceeds through multiple discrete intermediate conformational states, consistent with a rugged electromechanical energy landscape generated by electrostatic, hydrophobic, and steric interactions within the voltage-sensing domain (Lacroix and Bezanilla, 2011; Armstrong et al., 2016). Within this framework, positively charged arginine side chains are transiently stabilized near the CTC through sequential interactions with acidic countercharges and the conserved phenylalanine residue that forms the hydrophobic constriction site (Groome and Bayless-Edwards, 2020). Together, these interactions generate localized energy minima that slow gating-charge translocation through the HCS.

Experimental studies in the Shaker potassium channel further support this interpretation. Lacroix and Bezanilla (2011) demonstrated that the conserved phenylalanine residue (F290) controls transfer of the fourth gating charge (R4) across the voltage sensor. Their results indicate that F290 functions primarily as a component of the hydrophobic plug that shapes the local electric field and dielectric environment during gating. Although this work does not provide evidence for a direct Arg-Phe *π*–cation interaction, it establishes the critical structural role of the conserved phenylalanine in regulating gating-charge transfer.

Additional mechanistic insight was provided by Schwaiger et al. (2011), who used steered molecular dynamics simulations to investigate passage of the first translocating arginine (R4) through the hydrophobic constriction of the voltage-sensing domain. Their simulations demonstrated that voltage-sensor activation proceeds through discrete metastable intermediate conformations associated with sequential rearrangement of salt bridges and localized interactions between R4 and the conserved phenylalanine residue (F233). During barrier crossing, the aromatic ring of F233 undergoes a pronounced rotational “flip” that transiently opens the hydrophobic lock, permitting passage of the arginine side chain before returning to its original orientation. The simulations further showed that passage of R4 represents one of the major energetic barriers encountered during activation. Conversion of the upper S4 segment from an *α*-helical to a 3₁₀-helical conformation substantially reduced this barrier by facilitating predominantly translational movement of S4 while minimizing rotational rearrangements.

Importantly, Schwaiger et al. (2011) did not observe repetitive micro-oscillations or reversible back-and-forth movements of S4 during interaction with F233, nor did they quantify the lifetimes of the metastable intermediate conformations. Rather, their work demonstrates that gating-charge translocation occurs through a sequence of discrete intermediate states separated by local energetic barriers. Similarly, molecular dynamics simulations by Delemotte et al. (2011) support sequential charge-transfer steps through the HCS, with some of the slowest transitions occurring near the CTC because of local energetic constraints.

Within the present framework, these metastable intermediate states provide the structural basis for a theoretical extension of the gating mechanism. Under subthreshold depolarization, when the outward electric-field-driven force is insufficient to completely overcome the local barrier, a gating charge occupying a metastable intermediate may undergo localized reversible excursions within the confined HCS/CTC environment. Accordingly, the term stalling refers here to transient residence within a metastable intermediate state rather than complete cessation of S4 movement. Experimental observations of reversible gating currents under subthreshold depolarization (Armstrong and Bezanilla, 1973; Bezanilla, 2000), are consistent with bidirectional gating-charge movement and the existence of intermediate states, although they do not directly demonstrate the proposed local excursions.

The proposed micro-reversal corresponds to a sub-ångström (~0.1 Å) reversible displacement of an individual gating charge within the HCS, substantially smaller than the ~10 Å displacement associated with complete voltage-sensor activation. This value is not experimentally measured in NaV channels but represents an order-of-magnitude modeling estimate consistent with sub-ångström side-chain fluctuations observed in proteins on picosecond timescales (Zheng et al., 2006; Kwak et al., 2007). Likewise, the ultrafast timescales associated with the proposed vibrational dynamics are inferred from known molecular vibrational periods rather than direct observation of S4 micro-reversals. These parameters should therefore be regarded as physically motivated approximations intended to constrain the proposed mechanism rather than experimentally established quantities.

The concentrated electric field within the HCS is not proposed to generate molecular vibrations directly. Instead, it is expected to modulate intrinsic guanidinium vibrational modes through the vibrational Stark effect by shifting vibrational frequencies, polarizing the guanidinium charge distribution, and altering transition dipole moments. Consequently, any transient vibrational dynamics near the CTC are hypothesized to arise from the combined effects of localized S4 motion, transient interactions with countercharges and the conserved phenylalanine, changes in hydration, and the intense local electric field. Although neither Lacroix and Bezanilla (2011) nor Schwaiger et al. (2011) demonstrated direct *π*–cation interactions between the conserved phenylalanine and the arginine guanidinium group, such interactions remain chemically plausible based on established guanidinium–aromatic interaction motifs and cannot presently be excluded (Figure 2).

Finally, the proposed oscillatory electromechanical dynamics are restricted to voltage-sensor arginine gating charges undergoing incomplete translocation through the HCS/CTC region and are not expected to occur in generic arginine-containing proteins lacking the specialized voltage-sensor architecture, concentrated electric field, and metastable gating dynamics characteristic of NaV channels (Table 1). Although conserved arginine residues are present in other ion channels, including R163 in the AMPA receptor (Pokharna et al., 2025) and R76 in the glutamate-gated chloride channel (Lynagh et al., 2017), these proteins do not possess the S4 voltage-sensing mechanism responsible for voltage-dependent gating-charge translocation (Table 1). In the present framework, S4 gating charges are therefore unique because their voltage-dependent movements within the confined HCS/CTC environment are hypothesized to support transient electrodynamic interactions. Since each NaV channel contains four homologous voltage-sensing domains, a single channel may contain up to four potential electrodynamic emitters (Figure 1).

### 3.2 Intense electric field at the CTC creates conditions for coherent vibrational states

Within this highly localized electrostatic environment, the positively charged guanidinium group of arginine experiences strong forces that tend to align it along local electric field lines while simultaneously interacting with the *π*-electron system of the conserved phenylalanine. As the S4 segment approaches the CTC during depolarization, the interplay between electrostatic forces and *π*–cation interactions (Gallivan and Dougherty, 1999) can transiently stabilize intermediate conformations and generate steep local energy gradients. Consistent with this view, recent computational and experimental studies indicate that intermediate states of the S4 segment are dynamically regulated and stabilized by specific structural interactions, including transient salt-bridge networks and gating-charge interactions within the voltage-sensing domain (Biswas et al., 2026).

Accordingly, when the outward electric-field-driven force is insufficient to fully overcome the energetic barrier near the CTC, the S4 gating charge may undergo brief, small-amplitude reversible excursions (“micro-reversals”) around these metastable intermediate states. Within the proposed framework, such dynamics are characterized by rapid deceleration and re-acceleration of the arginine side chain within a spatially confined region near the HCS/CTC interface (Figure 2).

A high-resolution crystal structure of a bacterial NaV channel obtained by Payandeh et al. (2011) demonstrated that the conserved phenylalanine residue within the charge-transfer center (CTC) is strategically positioned such that S4 arginine side chains must pass in close proximity during gating charge translocation. This structural arrangement provides an opportunity for transient *π*–cation interactions to occur as the positively charged gating residues move through the voltage sensor domain. Notably, the arginine side chain contains a planar guanidinium group that exhibits amphiphilic properties, being relatively hydrophobic above and below its molecular plane while maintaining strong in-plane hydration through hydrogen bonding with water molecules or polar side chains (Armstrong et al., 2016). These unique physicochemical characteristics have been proposed to facilitate transient interactions between guanidinium groups and aromatic residues within proteins (Armstrong et al., 2016). Furthermore, molecular dynamics simulations employing forced S4 movement have shown that arginine guanidinium groups undergo coordinated sliding and rotational motions as they traverse the voltage sensor domain (Schwaiger et al., 2011).

Within the context of the present framework, these dynamic transitions may permit the guanidinium group to transiently sample *π*–cation configurations with the phenylalanine ring of the CTC (Schwaiger et al., 2011). Although the lifetimes of such configurations within NaV channels remain unknown, they could plausibly persist over timescales comparable to at least a few guanidinium vibrational periods (tens of femtoseconds) (Schwaiger et al., 2011). During these ultrafast intervals, the formation and relaxation of the interaction would involve rapid redistribution and acceleration of charge within the highly confined, high-electric-field environment of the CTC. We propose that the combined effects of transient stabilization at the CTC, repeated micro-reversals of S4 movement, and the intense local electric fields within the voltage sensor increase the effective dwell time of gating charges in this region. Consequently, these factors may enhance the probability of a shared temporal window during which multiple emitters occupy similar dynamical states, thereby creating conditions favorable for the emergence of cooperative electrodynamic phenomena (Table 2).

Under these conditions, arginine side chains may transiently exhibit partially phase-aligned vibrational dynamics. Although the extent to which such dynamics achieve true quantum coherence remains uncertain, this regime provides a physically plausible basis for enhanced electromechanical coupling and, potentially, short-lived cooperative emission among closely spaced NaV channels.

The rapid acceleration and deceleration of charge associated with these events can impulsively excite local vibrational modes of the guanidinium group. Ultrafast spectroscopic studies of molecular systems demonstrate that such impulsive perturbations can generate coherent vibrational wavepackets with well-defined phase relationships on femtosecond timescales (Mukamel, 1995; Hamm and Zanni, 2011). In this framework, the micro-reversal provides a temporal synchronization mechanism, while the intrinsic vibrational modes of the arginine side chain determine the characteristic frequencies of the resulting oscillations.

Note that the overlap between the timescale of proposed S4 micro-reversal dynamics (sub-picosecond) and that of arginine vibrational modes (tens of femtoseconds) allows multiple vibrational cycles to occur during a single reversal event.

Importantly, the HCS should not be regarded as an environment that prevents decoherence. The dominant sources of decoherence in the proposed system are expected to include thermal fluctuations, coupling to surrounding protein vibrational modes, electrostatic noise arising from charged residues and membrane-potential fluctuations, and residual interactions with nearby solvent molecules. These processes would be expected to rapidly disrupt phase relationships and broaden transition frequencies. Consequently, any coherent or partially phase-correlated dynamics are likely to be extremely short-lived and confined to sub-picosecond timescales.

Because the HCS is relatively dehydrated and geometrically constrained, coupling to environmental degrees of freedom may be modestly reduced compared with fully solvated protein environments. However, this reduction is not expected to eliminate decoherence. Rather, it may permit transient vibrational correlations to persist for durations approaching the upper range of experimentally observed vibrational dephasing times in proteins, while still decaying rapidly on timescales of approximately 0.1–1 ps (Zheng et al., 2006; Kwak et al., 2007).

Moreover, in the presence of intense electric field near the CTC, the Stark effect enhances polarization of the guanidinium group, which carries a delocalized positive charge, thereby increasing its effective electric dipole moment. This, in turn, can facilitate more pronounced acceleration of the arginine side chain during micro-reversal. In addition to promoting vibrational excitation, such rapid charge acceleration increases the instantaneous dipole acceleration, a necessary condition for electromagnetic radiation (Feynman et al., 1963; Jackson, 1999).

Under non-equilibrium conditions generated by membrane depolarization and transient S4 gating dynamics, coherent vibrational excitation may transiently enhance coupling between oscillating molecular dipoles and the electromagnetic field (Mukamel, 1995), potentially increasing the probability of higher-order radiative processes such as two-photon emission. However, two-photon emission is intrinsically a second-order process and is therefore expected to occur with substantially lower probability than single-photon emission (Mandel and Wolf, 1995).

Taken together, the intense electric field, nanoscale confinement, and barrier-induced micro-reversal dynamics at the CTC create a physically plausible setting in which non-equilibrium excitation of coherent vibrational states can occur. These states provide the immediate dynamical substrate for any subsequent radiative processes, while remaining strongly constrained by rapid decoherence and dominant non-radiative relaxation in the protein environment (Table 3).

**Table 3:** Proposed conditions required for Dicke-type cooperative emission in the present NaV-channel framework.

Requirement for cooperative emission	Proposed realization in the present framework	Current status
Subwavelength ensemble size	The AIS diameter (~0.5–1 μm) is substantially smaller than the predicted mid-infrared wavelength (~3–10 μm), allowing neighboring emitters to interact within approximately the same electromagnetic phase.	Supported by known AIS geometry
Common excitation	Shared subthreshold membrane depolarization provides a common driving field for neighboring S4 voltage sensors.	Supported by neuronal physiology
Near-synchronous activation	Neighboring NaV channels may undergo activation within a narrow temporal window during synchronized synaptic input.	Proposed
Similar transition frequencies	Comparable local electric fields within neighboring HCS/CTC regions may produce similar vibrational transition frequencies.	Proposed
Partial dipole alignment	Similar orientation of S4 voltage sensors within the membrane may promote partial alignment of transition dipoles.	Proposed
Dense emitter population	The AIS contains approximately 10^4^–10^5^ NaV channels, providing a sufficiently large ensemble for possible collective effects.	Supported by experimental studies
Transient phase correlations	Sub-picosecond S4 micro-reversal dynamics may permit short-lived phase correlations before environmental decoherence dominates.	Proposed
Coherence time exceeding emission timescale	The proposed coherence window (~0.1–1 ps) is hypothesized to be sufficient for transient cooperative emission if the above conditions are satisfied.	Order-of-magnitude estimate
Weak environmental decoherence	Reduced hydration within the HCS may modestly decrease environmental coupling, although thermal fluctuations, protein vibrations, and electrostatic noise are expected to rapidly limit coherence.	Supported qualitatively; quantitative evaluation required
Collective radiative enhancement	If these conditions are simultaneously satisfied, emission intensity could scale more strongly than that of independent emitters, analogous to Dicke-type cooperative emission.	Central hypothesis; requires computational and experimental validation

### 3.3 Electric field–mediated facilitation of cooperative emission at the AIS

#### 3.3.1 Dicke-type cooperative emission

Dicke-type radiation, or superradiance, describes a cooperative emission process in which an ensemble of closely spaced emitters interacts through a shared electromagnetic field mode, resulting in collective rather than independent radiation (Dicke, 1954; Gross and Haroche, 1982) (Refer to Table 3 for proposed conditions required for Dicke type cooperative emission in the proposed NaV channel network). When subsets of emitters exhibit sufficiently similar transition frequencies and transient partial phase alignment, their dipole moments may evolve in a correlated manner, enhancing the radiative decay rate. In contrast to independent emission, which scales linearly with the number of emitters (∝ N), cooperative emission may scale superlinearly, approaching ∝ *N*^2^ under idealized conditions.

In biological systems, strong decoherence and structural heterogeneity are expected to limit both the duration and extent of Dicke-type cooperative behavior. Nevertheless, cooperative emission sufficiently robust to persist under thermal conditions has been reported to enhance quantum yield in extended networks of tryptophan chromophores within taxol-stabilized microtubules in aqueous solution at room temperature (Babcock et al., 2024). While the physical mechanism proposed here is distinct, these findings suggest that transient cooperative electrodynamic effects may, in principle, arise in warm and dissipative biological environments. Accordingly, transient and spatially restricted cooperative interactions may occur within densely packed and geometrically organized ensembles—such as NaV channels at the AIS—where emitters share similar orientations and experience a common electromagnetic environment within the HCS under near-threshold membrane conditions.

During a coherence window (~0.1–1 ps), each emitter may undergo multiple rapid, small-amplitude reversible fluctuations (“micro-reversals”) near the CTC (Figure 2). These oscillatory micro-reversals do not increase the number of physical emitters, but instead increase the number of radiatively active transitions occurring within the same temporal interval. Thus, if each of N emitters undergoes m micro-reversal cycles, the effective number of emission opportunities scales as mN. This should be interpreted as a temporal amplification mechanism in which repeated, partially phase-correlated oscillations contribute collectively to a shared electromagnetic field, rather than as an increase in independent emission sources.

If transient partial phase alignment is maintained within this interval, the resulting emission may exhibit cooperative enhancement consistent with Dicke-type behavior, with intensity scaling approaching ∝ (mN)^2^ under idealized conditions. However, such coherence is expected to remain highly transient, and any cooperative enhancement should therefore be regarded as partial and short-lived. Within the present framework, Dicke-type emission represents a constrained and experimentally testable phenomenon that does not require sustained or long-range quantum coherence.

#### 3.3.2 Cooperative enhancement of photon emission at the axon initial segment

The axon initial segment (AIS) exhibits a highly ordered nanoscale architecture characterized by a membrane-associated periodic skeleton (MPS) composed of actin rings spaced at ~190 nm and interconnected by spectrin tetramers (Leterrier, 2018). Voltage-gated sodium (NaV) channels are anchored to this scaffold through ankyrin-G and βIV-spectrin interactions, producing a quasi-periodic spatial organization (Xu et al., 2013). Such structural organization suggests that local subsets of NaV channels—particularly NaV1.6—may share similar orientations relative to the membrane electric field.

Near threshold, NaV channels may activate in temporally clustered fashion, bringing their S4 segments into proximity with the CTC within a narrow temporal interval. Because traversal of the CTC involves overcoming a local energy barrier, S4 motion is expected to transiently slow, increasing residence time in intermediate metastable states. Under these conditions, small-amplitude ultrafast reversible fluctuations (“micro-reversals”) of gating charges may occur.

Although the dwell time of S4 arginine residues at the CTC has not been directly measured, gating-current kinetics and molecular simulations suggest dynamics occurring on picosecond-to-nanosecond timescales (Bezanilla, 2000; Delemotte et al., 2011; Schwaiger et al., 2011). During these intervals, the macroscopic membrane potential remains effectively constant, defining a short coherence window (*τ* ≈ 0.1–1 ps) comparable to vibrational dephasing times observed in proteins (Zheng et al., 2006; Kwak et al., 2007). Under near-threshold conditions, oscillating arginine side chains (“emitters”) experience a shared high-intensity electric field (~10^7^–10^8^ V/m) within the HCS, potentially promoting partial dipole alignment and transient phase correlations during this coherence window.

At the level of individual channels, any radiative emission associated with rapid charge reorientation is expected to be extremely weak and spatially localized. However, the dense clustering of NaV channels at the AIS creates conditions in which subsets of emitters may interact within a shared electromagnetic environment. From the perspective of the electromagnetic field, these oscillators may become only partially distinguishable, permitting weak electrodynamic coupling through common field modes.

Because the diameter of the AIS (~0.5–1 μm) is smaller than typical mid-infrared wavelengths (~3–10 μm), the emitter ensemble occupies a subwavelength volume. Under these conditions, emitters experience a nearly uniform electromagnetic phase across the AIS cross-section, facilitating coupling to a shared field mode. This geometric constraint may therefore support weak field-mediated interactions capable of transient cooperative emission.

At the same time, the circular cytoskeletal organization of the AIS is unlikely to support complete phase alignment of emitters across the entire structure, as dipole orientations are expected to vary with angular position. Instead, the proposed mechanism is more likely to involve localized subensembles of emitters that share similar microenvironmental conditions and experience comparable local electric fields. Within these restricted domains, transient phase alignment could, in principle, be sufficient to produce weak cooperative emission over brief time intervals. Accordingly, any radiative enhancement would be expected to arise from spatially localized, anisotropic clusters rather than from a globally phase-coherent ensemble spanning the entire AIS.

Note that other voltage-gated ion channels present at the AIS, such as Kv7.2/7.3 potassium channels, exhibit distinct voltage-sensor kinetics and are therefore unlikely to become temporally synchronized with NaV channels (Leterrier, 2018). Moreover, similar transition frequencies of emitters are a requirement for cooperative emission, which will be unlikely for emitters within structurally different membrane proteins. Similarly, channels located in the soma and dendrites lack the periodic nanoscale organization characteristic of the AIS, reducing the likelihood of comparable cooperative effects in those regions.

In addition, the AIS differs substantially from other neuronal compartments, including the soma and dendrites, in ways that may favor transient electrodynamic coupling. The AIS possesses a relatively low membrane capacitance, the highest density of voltage-gated sodium channel, and strong enrichment of the NaV1.6 isoform, which exhibits rapid activation kinetics (Hu et al., 2009; Kole and Stuart, 2012). Together, these properties permit small-amplitude, sub-millisecond membrane-potential fluctuations near threshold that may favor ultrafast S4 voltage-sensor dynamics within the CTC.

As discussed earlier, the intense electric field within the CTC is expected to enhance polarization of the arginine guanidinium group, thereby increasing its effective transition dipole moment. Through the Stark effect, this field may partially homogenize vibrational transition frequencies and promote preferential alignment of oscillating dipoles along a common field direction, creating conditions more favorable for transient cooperative emission. However, the Stark effect alone is insufficient to generate phase coherence.

Within the present framework, transient phase alignment is more plausibly driven by near-synchronous excitation of closely spaced NaV channels arising from shared membrane-potential dynamics at the AIS. Photon-mediated dipole–dipole interactions may further contribute to weak phase coupling, although such interactions are expected to remain strongly constrained by rapid decoherence in the biological environment. Consequently, any enhancement of phase alignment and cooperative behavior would likely remain highly transient and restricted to brief intervals of synchronous subthreshold activation at the AIS.

The proposed physical conditions that may permit transient cooperative electrodynamic interactions among neighboring NaV-channel emitters are summarized in Table 2. These conditions are derived from established principles of cooperative emission together with known structural and biophysical properties of the axon initial segment and are presented as experimentally testable criteria rather than established characteristics of NaV-channel function.

Accordingly, we propose a testable hypothesis in which subthreshold membrane fluctuations at the AIS permit transient, weakly coherent electrodynamic coupling among subsets of NaV channels, giving rise to short-lived correlations in emitted infrared photons or associated charge oscillations. Importantly, this framework does not require sustained quantum coherence or robust multipartite entanglement but instead focuses on transient mesoscale electrodynamic correlations that may arise under biologically realistic conditions.

#### 3.3.3 Cooperative coupling of emitters at clusters of axon initial segments

Layer IV is among the most densely populated regions of the neocortex (Rockel et al., 1980; Douglas and Martin, 2004). Given the close spacing of neurons within cortical minicolumns (Mountcastle, 1997; Buxhoeveden and Casanova, 2002), neighboring axon initial segments (AISs) may, in some cases, be separated by distances on the order of the wavelength of the proposed mid-infrared emissions. Such spatial proximity could, in principle, increase opportunities for localized electromagnetic interactions between neighboring AISs.

Within the present framework, if transient mid-infrared cooperative emission events occur, the resulting near-field electromagnetic interactions could provide a mechanism for weak electrodynamic coupling between emitters associated with different neurons within the same cortical minicolumn. Although highly speculative, such local interactions may transiently enhance the temporal coordination of neuronal activity among neighboring neurons without replacing established synaptic or network mechanisms. This possibility may be particularly relevant in layer IV of the primary visual cortex, where densely packed neurons receive synchronized thalamocortical inputs representing adjacent regions of visual space. Any such electrodynamic interactions, if they occur, would therefore complement the classical integration of thalamocortical inputs during early cortical sensory processing rather than constitute an alternative mechanism.

## 4 Evaluation of photon emission by vibrating arginine side chains during S4 reversal

We first estimate the radiated energy associated with positively charged arginine side chains undergoing micro-reversal dynamics within the HCS. Since the guanidinium group of the arginine side chain represents an extended molecular charge distribution with a delocalized positive charge, rather than a point charge, its radiative properties are more appropriately described using a dipole-radiation framework. Experimental infrared studies of guanidinium-containing vibrational modes have demonstrated finite transition dipole moments associated with CN stretching vibrations of the arginine side chain (Barth, 2007; Hamm and Zanni, 2011). As indicated in Equation 1, the vibrating guanidinium group is modeled as an oscillating electric dipole characterized by a time-dependent dipole moment:


$$p(t)=p_{o}\cos(\mathrm{\Omega t})$$ (1)

where ( $p_{o}$ ) is the effective dipole amplitude and Ω is the angular frequency of the vibrational mode.

The cycle-averaged radiated power from an oscillating dipole is given by


$$P=\frac{\Omega ⁴p_{0}^{2}}{12\pi \varepsilon_{o}c^{3}}$$ (2)

where $\varepsilon_{o}$ is the permittivity of free space and (c) is the speed of light. Using Ω ≈ 3.0 × 10^14^ s^−1^, corresponding to the arginine guanidinium vibrational band near 1,600 cm^−1^, and adopting representative transition dipole moments reported for protein vibrational modes, the resulting radiative power remains extremely small. Consequently, the energy emitted during a coherence interval of *τ* ≈ 0.1–1 ps is expected to be many orders of magnitude smaller than the energy of a mid-infrared photon.

These estimates suggest that isolated guanidinium vibrational emitters are unlikely to generate experimentally detectable radiation. Rather, the calculations indicate that if photon emission associated with S4 dynamics were to become observable, some form of cooperative enhancement among multiple emitters would likely be required. We, therefore, next examine how collective behavior could influence photon yield in ensembles of oscillating arginine side chains.

Accordingly, we propose a testable hypothesis: subthreshold membrane fluctuations at the AIS may permit transient electrodynamic coupling among subsets of NaV channels, giving rise to short-lived phase correlations in emitted infrared photons or associated charge oscillations. Importantly, this framework does not require long-lived quantum coherence or robust multipartite entanglement, but instead focuses on transient mesoscale correlations that may arise under biologically realistic conditions.

If such phase correlations are established, cooperative radiative effects could, in principle, emerge. For an ensemble of (N) emitters, each undergoing (m) micro-reversal cycles within the coherence interval, the total number of radiatively active transitions scales as (mN). As indicated in Equation 3, in the incoherent limit, the total emitted power scales linearly with the number of transitions.


$$P_{\mathrm{tot}}\propto \mathrm{mN}$$ (3)

However, in the presence of phase coherence within the HCS, cooperative emission may scale superlinearly as


$$\;P_{\mathrm{tot}}\propto {\left(\mathrm{mN}\right)}^{2}\;$$ (4)

consistent with Dicke-type collective dipole emission (Dicke, 1954; Gross and Haroche, 1982).

Based on Equation 2, the single-emitter photon yield ( Nγ(1) ) during a coherence interval ( τ ) is given by


$$\mathrm{N\gamma}(1)=\frac{\;\Omega ⁴p_{0}^{2}\tau }{12\pi \varepsilon_{o}c^{3}\mathrm{h\nu}}$$ (5)

where (h) is Planck’s constant and ( ν ) is the emission frequency.

Under cooperative conditions, the photon yield ( Nγ(coop) ) scales according to


$$\mathrm{N\gamma}(\text{coop})\approx {\left(\mathrm{mN}\right)}^{2}\;N_{\gamma }(1)$$ (6)

Substituting the single-photon emission condition under cooperative emission into Equation 6 yields Equation 7.


$$1\approx {\left(\mathrm{mN}\right)}^{2}\mathrm{N\gamma}(1)$$ (7)

Solving Equation 7 for the ensemble size required to produce approximately one photon per coherence interval yields:


$$N\approx \frac{1}{\left[m\surd \mathrm{N\gamma}(1)\right]}\;$$ (8)

Substituting Equation 5 into Equation 8 yields:


$$N\approx \frac{1}{m}\times \surd [\;\frac{12\pi \varepsilon_{o}c^{3}\mathrm{h\nu}}{\;\Omega ⁴p_{0}^{2}\tau }]$$ (9)

Approximate values for most variables in Equation 9 are available, with the notable exception of ( $p_{o}$ ). Since the effective transition dipole moment of the guanidinium vibrational mode within the HCS has not been experimentally determined, estimates of the required ensemble size should presently be interpreted parametrically. Future infrared spectroscopic studies or QM/MM simulations may help constrain plausible values of ( $p_{o}$ ) and refine these predictions.

Finally, the intense electric field within the HCS may induce additional polarization of the guanidinium charge distribution and modify the effective transition dipole moment through a vibrational Stark effect. Because radiative power scales with the square of the oscillating dipole moment (Equation 2), field-induced enhancement of (p_0) could strengthen radiative coupling among neighboring emitters and reduce the ensemble size required for cooperative photon emission. The magnitude of this effect remains uncertain, however, and would require dedicated QM/MM calculations or spectroscopic investigation.

### 4.1 Estimation of the number of micro-reversals per coherence period

The estimate of m∼10−50 micro-reversals per coherence window arises from the ratio of two characteristic timescales: the coherence lifetime of vibrational modes in proteins (~0.1–1 ps) and the intrinsic period of arginine guanidinium vibrational modes (~10–30 fs). This yields a theoretical range of approximately 5–50 oscillatory cycles. The lower bound reflects rapid decoherence or overdamped dynamics, whereas the upper bound corresponds to weakly damped oscillations within the hydrophobic constriction site, where reduced dielectric screening and limited hydration may transiently prolong coherence. The exact value of m is therefore expected to depend on the local energy landscape at the charge-transfer center and on membrane potential fluctuations that can repeatedly re-drive S4 motion.

### 4.2 Demonstration of principle using a point-charge approximation

To illustrate the magnitude of enhancement that cooperative emission could provide, we next perform an order-of-magnitude calculation using a hypothetical oscillating point charge in place of the guanidinium dipole within the HCS. As discussed in the previous section, the guanidinium group is more appropriately described as an oscillating dipole rather than a point charge. The present analysis is therefore intended solely as a demonstration of principle to estimate the ensemble sizes that might be required if cooperative radiative effects were present.

The guanidinium CN vibrational band of arginine lies near a wavenumber of approximately $1,600\;{\mathrm{cm}}^{-1}$ , corresponding to a vibrational frequency of $\approx 4.8\times 10^{13}$ , and an angular frequency of $\Omega =2\pi \nu \approx 3.0\times 10^{14}\;s^{-1}.$

Assuming an oscillation amplitude for the hypothetical point charge comparable to that proposed for guanidinium vibrational motion (Zheng et al., 2006; Kwak et al., 2007), $x_{0}∼0.01–0.1\;\overset{´}{A}.$

The peak acceleration can be estimated as


$$a∼\Omega^{2}x_{0}\approx 10^{17}–10^{18}\;m\;s^{-2}$$ (10)

Using the classical Larmor formula, the radiated power from a single oscillating point charge is given by


$$P=\frac{q^{2}a^{2}}{6\pi \varepsilon_{0}c^{3}}$$ (11)

where q ≈ e is the effective charge. Substituting the values from Equation 10 into Equation 11 yields an estimated radiated power per emitter of


$$P∼10^{-20}–10^{-18}\;W$$ (12)

The energy of a single photon corresponding to the guanidinium vibrational frequency is


$$E_{\gamma }=\mathrm{h\nu}\approx 3.2\times 10^{-20}\;J\;$$ (13)

The radiated energy of the point-charge approximation of the guanidinium group during the proposed coherence interval of 0.1–1 ps, calculated using Equation 12, is:


$$E_{\mathrm{rad}}\approx \mathrm{P\tau}∼10^{-33}–10^{-30}\;J\;$$ (14)

After substituting the values obtained from Equations 13 and 14 into Equation 15, the corresponding number of photons emitted per burst event by a single hypothetical point-charge emitter is:


$$N_{\gamma }^{\left(1\right)}\approx \frac{E_{\mathrm{rad}}}{E_{\gamma }}∼10^{-13}–10^{-10}\;$$ (15)

Thus, photon emission from a single point-charge approximation of the guanidinium ion would be effectively undetectable. However, assuming cooperative emission among multiple emitters, the resulting radiated signal could be enhanced, as described below. Assuming cooperative emission over the transient coherence interval (*τ* = 0.1–1 ps), the ensemble size required to generate approximately one photon per cooperative burst is estimated to be on the order of $10^{3}-10^{5}$ emitters, based on Equation 8 and assuming *m* = 10–50 micro-reversal cycles per emitter.

Because the AIS contains approximately $10^{4}–10^{5}$ NaV channels (Kole et al., 2008), these estimates suggest that the required ensemble sizes are, in principle, compatible with the number of available channels within the AIS. If each of the four homologous domains of a NaV channel were capable of contributing an emitter during a cooperative event, the total number of emitted photons could potentially reach approximately 4–40 photons across the entire AIS during a single cooperative interval of 0.1–1 ps.

The frequency of such cooperative events would be expected to depend strongly on the subthreshold electrodynamic state of the AIS, which in turn may be influenced by factors such as the rate of membrane depolarization, the spatiotemporal organization of synaptic inputs, and the distribution of NaV channel activation states. Consequently, the overall rate of photon emission would likely vary considerably over time.

Since cooperative emission events are hypothesized to occur only during specific phases of subthreshold input integration at the AIS, rather than throughout the entire period of neuronal activation, the total number of photons emitted by a neuron is expected to depend on the frequency of such events. If each cooperative event produces approximately *n* photons, the photon emission rate can be approximated as $k\times \frac{n\;\text{photons}}{\text{Cooperative event}}$ , where k represents the frequency of cooperative emission events arising from S4 micro-reversal dynamics during subthreshold depolarization at the AIS.

Accordingly, infrared photon emission in the present framework is predicted to be *episodic* rather than *continuous*, with the overall emission rate depending on the rate of cortical neuronal activation and the spatiotemporal organization of synaptic inputs that determine the duration of subthreshold integration. Importantly, once the membrane potential at the AIS reaches action potential threshold, S4 segments are expected to complete their forward movement rapidly, with little opportunity for the micro-reversal dynamics proposed to underlie cooperative emission. Consequently, infrared photon generation is not expected during the action potential itself but rather during the preceding subthreshold phase of synaptic integration.

On this basis, it is tentatively speculated that infrared photon emission rates at individual AISs may fall within the range of hundreds of photons per second. However, this estimate remains highly provisional, as several parameters—including the probability of cooperative emission, the effective ensemble size, the duration of coherence intervals, the number of cooperative events occurring during input integration, and the firing rates of cortical neurons—have not yet been experimentally determined and will require further theoretical modeling and experimental investigation.

Importantly, the reader should recognize that the point-charge approximation employed in this section almost certainly overestimates the radiative output of individual emitters. As discussed in Section 4.1.1, the guanidinium group is more accurately represented as an oscillating dipole with a delocalized charge distribution. Accordingly, the true single-emitter photon yield is expected to be substantially lower, implying that larger ensemble sizes may be required for cooperative emission under physiologically realistic conditions.

### 4.3 High density of NaV channels at the AIS contributes to cooperative emission

Given that the AIS contains on the order of $10^{4}–10^{5}$ NaV channels (Kole et al., 2008), with each channel potentially contributing up to four emitters through its four homologous S4 domains, these estimates suggest that cooperative effects could, in principle, enhance photon emission to a potentially detectable level. Furthermore, note that each NaV channel comprises four homologous domains (DI – DIV), each containing an S4 segment with gating charges. Accordingly, each channel can potentially contribute multiple potential emitters.

Owing to the cylindrical geometry of the AIS membrane, these emitters are distributed around the axonal circumference, with subsets sharing similar orientations relative to the local electric field. Such partial orientational alignment may facilitate cooperative interactions within localized regions, although full global alignment across the entire circumference is unlikely.

Finally, the decay of such transiently phase-correlated vibrational states may proceed via single- or multi-photon pathways. While one-photon emission is expected to dominate, the combination of strong local electric fields and dipole alignment may, in principle, permit higher-order processes such as two-photon emissions. Moreover, the relative likelihood of these pathways remains uncertain and will require further theoretical and experimental investigation.

## 5 Biophoton emission in biological systems

Ultraweak photon emission (“biophotons”) has been experimentally observed in a variety of biological systems, including brain tissue, and is generally attributed to oxidative metabolic processes that generate electronically excited molecular species (Popp et al., 1994; Isojima et al., 1995; Kobayashi et al., 1999a; Kobayashi et al., 1999b; Cifra et al., 2011). These emissions are typically broadband, incoherent, and arise predominantly within the visible to near-infrared spectral range as a consequence of electronic-state transitions. In contrast, the present work considers a distinct and previously unexplored regime of vibrationally mediated electromagnetic emission in the mid-infrared range associated with rapid conformational dynamics of molecular structures rather than electronic excitation.

Importantly, Isojima et al. (1995) demonstrated depolarization-induced biochemiluminescence in hippocampal tissue that was suppressed by tetrodotoxin, suggesting that the observed photon emission was indirectly linked to neuronal metabolic activity associated with membrane excitation. Although these emissions were interpreted within a metabolic framework, the findings nevertheless support the broader principle that neuronal activity can be associated with detectable ultraweak photon emission.

Interestingly, previous work predicted the release of infrared photons during cortical information processing as a consequence of information compression (Stoll, 2024b). Within that thermodynamic framework, the reduction of uncertainty in a putative quantum computational process is associated with spontaneous free-energy release, which may contribute to synchronized firing of statistically distributed neuronal ensembles. Although the mechanistic assumptions differ from those of the present model, the prediction of infrared-range energetic signatures during cortical processing provides an intriguing point of convergence with the present speculation regarding quantum processes associated with S4 voltage-sensor dynamics.

The apparent absence of robust experimental detection of endogenous mid-infrared biophotons in neural tissue is likely attributable to the strong absorption of mid-infrared radiation by water, which dominates the intracellular environment and severely attenuates electromagnetic propagation within this spectral range (Guo et al., 2011). Consequently, any such emissions would be expected to remain highly localized, ultrafast, and difficult to detect compared with the relatively more accessible visible range biophoton signals.

Importantly, previous biophoton studies have primarily focused on incoherent metabolically generated emissions arising from electronic-state transitions, whereas the present hypothesis concerns transient, vibrationally driven, and potentially correlated infrared emission arising from electromechanical protein dynamics. Thus, the proposed mechanism represents a fundamentally different physical regime of photon generation. Supporting the broader relevance of this spectral domain, externally applied mid-infrared radiation has been shown to modulate neural activity, including membrane excitability and neuronal firing (Wells et al., 2005; Zhang et al., 2021; Liu et al., 2025), indicating that neural systems are responsive to electromagnetic interactions within the mid-infrared range.

Taken together, these observations motivate a testable hypothesis in which endogenous mid-infrared emission arising from rapid conformational dynamics of voltage-gated sodium channel contributes to localized electromagnetic interactions within the membrane environment. This framework does not invoke long-range photon signaling, but instead proposes a short-range, ultrafast, and dissipative regime of electromechanical–electromagnetic coupling. In this sense, neurons may be viewed not only as electrical signaling units, but also as systems in which molecular-scale electromagnetic processes coexist with classical electrophysiology, although this possibility remains speculative and experimentally unverified.

## 6 Transient non-classical correlations and their relation to neural information processing

In the proposed framework, synaptic inputs arriving at a multipolar neuron are hypothesized to become transiently reflected in phase-correlated electrodynamic states of emitters at the AIS (Refer to Table 4 for proposed events). Inputs from long-range cortical circuits, which carry information associated with the brain’s interaction with external stimuli, are predominantly excitatory. Activation of these synapses generates local currents that converge onto the AIS, thereby modulating its membrane potential (Figure 3). In this manner, temporally synchronized synaptic activity determines the evolution of membrane state and local electromagnetic environment surrounding NaV channels at the AIS.

**Table 4:** Proposed sequence of molecular and electrodynamic events underlying the hypothesis.

Step	Proposed event	Status
1	Synchronized excitatory synaptic inputs converge onto the AIS and generate transient subthreshold membrane depolarization.	Established neurophysiology
2	Subthreshold membrane-voltage fluctuations modulate the probability and timing of S4 voltage-sensor movement within NaV channels.	Supported by gating-current studies
3	Near the CTC/HCS, S4 gating charges transiently occupy metastable intermediate states.	Supported by structural and functional studies
4	During subthreshold activation, some gating charges may undergo localized sub-ångström reversible excursions (“micro-reversals”).	Proposed extension
5	Rapid acceleration and deceleration of arginine guanidinium groups excite oscillatory dipole dynamics.	Proposed extension
6	Dense clustering and ordered organization of NaV channels at the AIS expose neighboring emitters to similar local electric fields and membrane-voltage fluctuations.	Supported by AIS structural studies
7	Neighboring emitters may transiently exhibit similar transition frequencies, partially aligned dipole orientations, and short-lived phase correlations during sub-picosecond coherence windows.	Proposed extension
8	Under these conditions, weak cooperative electrodynamic interactions analogous to Dicke-type collective emission may transiently occur, enhancing otherwise negligible infrared emission.	Proposed extension
9	Rapid environmental decoherence terminates the transient cooperative state within approximately 0.1–1 ps.	Supported by protein vibrational dephasing studies and theoretical interpretation
10	Any emitted mid-infrared photons or cooperative photon statistics constitute experimentally testable predictions of the framework.	Testable prediction

**Figure 3:**
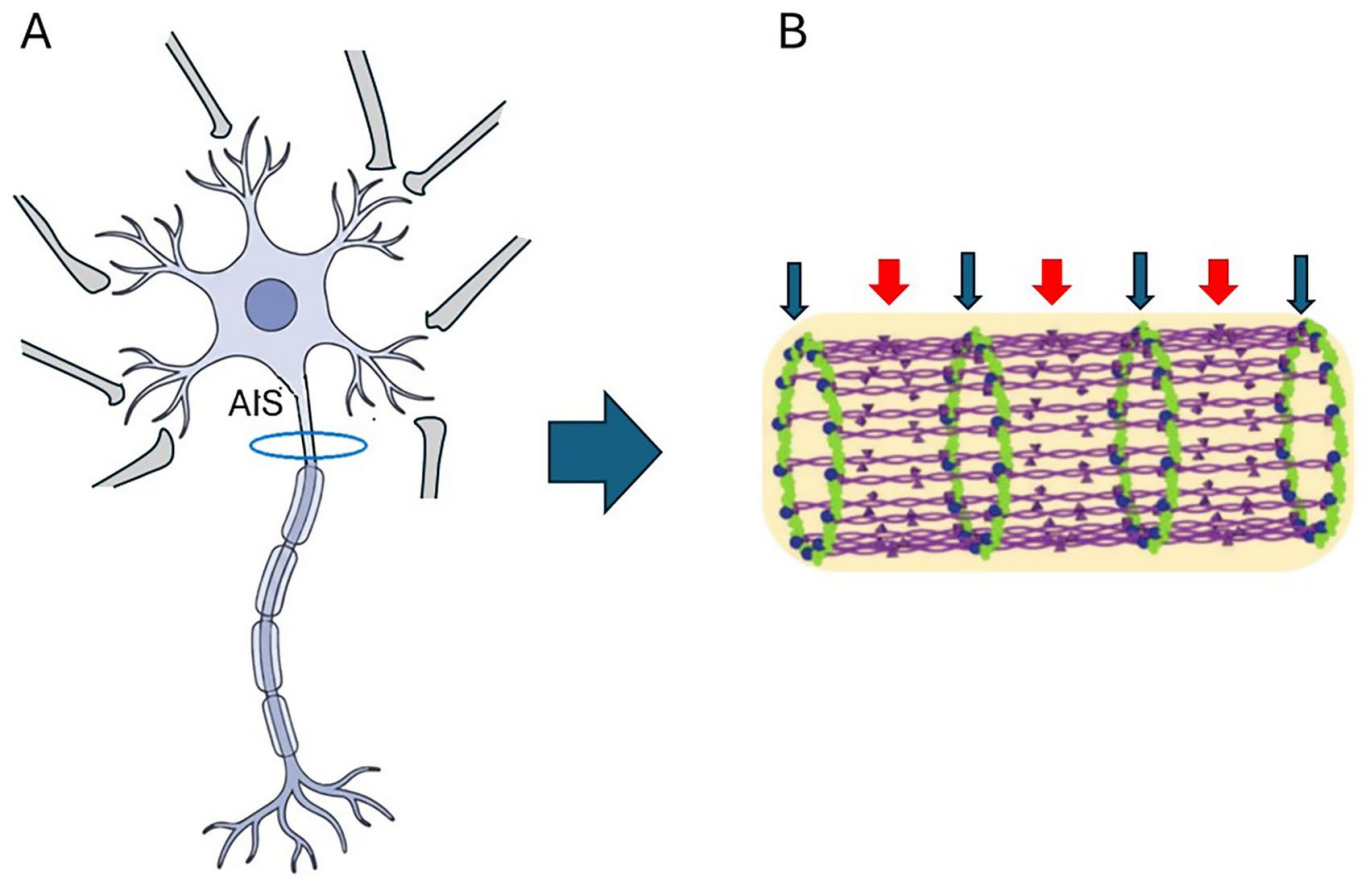
Organization and functional architecture of the axon initial segment (AIS). **(A)** The axon initial segment (AIS) integrates synchronous synaptic inputs arriving at the soma. These inputs generate local membrane currents (illustrated by green arrows surrounding the soma) that propagate along the somatic membrane and converge onto the AIS. Consequently, the membrane potential at the AIS reflects the integrated influence of synaptic inputs originating from distributed brain regions. As the AIS membrane potential approaches the threshold for action potential initiation, NaV channel S4 voltage sensors enter a near-threshold or subthreshold dynamical regime. Under these conditions, S4 segments may transiently stall and undergo rapid micro-reversal dynamics near the charge-transfer center (CTC), creating conditions proposed to favor transient electrodynamic coupling and cooperative emission. The sodium channel isoform NaV1.6 is preferentially enriched at the AIS, particularly within the distal AIS, which serves as the principal site of action potential initiation. **(B)** A magnified schematic of the AIS cytoskeletal architecture illustrates the periodic ring-like arrangement of actin filaments (green) interconnected by spectrin tetramers (magenta). Voltage-gated sodium (NaV) channels are anchored to this actin–spectrin scaffold through ankyrin-G interactions (indicated by red arrows), producing a quasi-periodic lattice-like organization along the axonal membrane. Super-resolution imaging studies have shown that NaV channels exhibit an approximate periodic spacing of ~190 nm coordinated by this underlying cytoskeletal structure. The total number of NaV channels within the AIS is estimated to be on the order of 10^4^–10^5^ channels per neuron, potentially providing a sufficiently large ensemble of emitters for transient collective electrodynamic effects. Under subthreshold membrane conditions associated with temporally synchronized synaptic activity, subsets of S4 segments may transiently occupy metastable states near the CTC and undergo rapid micro-reversal oscillations. Within the present framework, the positively charged arginine side chains of the S4 gating segments, oscillating within the intense local electric field of the CTC/hydrophobic constriction site (HCS), are proposed to function as emitters capable of generating weak infrared photon emission through a transient Dicke-type cooperative emission mechanism. Panel **(B)** illustration is modified from the periodic axonal cytoskeleton model reported by Xu et al. (2013).

The resulting membrane-potential dynamics may, in turn, influence the dwell time and electromechanical behavior of S4 voltage-sensor segments near the CTC. Under near-threshold conditions, the probability of transient interactions between S4 gating charges and the CTC/HCS is expected to increase. Subthreshold depolarization may therefore modulate the likelihood of S4 micro-reversal events, the degree of transient phase alignment among neighboring emitters within the HCS, and the probability of cooperative electromagnetic emission. Consequently, synchronized synaptic activity may transiently shape collective electrodynamic dynamics at the AIS without requiring sustained quantum coherence or long-lived entanglement.

Within this framework, transient cooperative states are proposed to emerge among ensembles of emitters during brief stalling of S4 motion at the CTC under subthreshold membrane voltages (Table 4). The intense local electric field within the HCS may transiently support ultrafast vibrational coherence during this dwell interval. Because S4 segments undergo coupled rotational and translational motion, neighboring emitters exposed to similar membrane potentials along the AIS may tend to adopt partially aligned orientations relative to the local electric field. In addition, rapid micro-reversal oscillations may increase the probability of transient cooperative electrodynamic coupling among nearby emitters. Experimental detection of mid-infrared photon emission exhibiting signatures consistent with superradiance would support the presence of transient cooperative electrodynamic correlations arising from these collective dynamics.

Synaptic activity also generates graded spatial variations in membrane potential along the proximal-to-distal axis of the AIS. During depolarizing synaptic input, for example, membrane potential at proximal regions of the AIS may approach action-potential threshold earlier than more distal regions, potentially reducing the probability or amplitude of S4 micro-reversal dynamics (m) in those local domains. Within this framework, transient non-classical correlations among S4-associated emitters may therefore encode information related to the integrated synaptic input state of the neuron (Figure 3). Importantly, membrane-potential fluctuations associated with synaptic integration occur on sub-millisecond timescales (Kole et al., 2007), whereas the proposed cooperative electrodynamic events are confined to sub-picosecond coherence windows. Accordingly, during any individual cooperative-emission interval, the local membrane potential of the AIS can be considered effectively constant.

In the present model, transient cooperative correlations are proposed to arise from membrane-voltage fluctuations generated by synchronized synaptic activity. Synaptic inputs may therefore serve as a gating mechanism through which information associated with cortical processing and environmental interaction becomes transiently encoded in the collective electrodynamic state of NaV channel ensembles at the AIS. The proposed non-classical phase correlations among emitters do not necessarily imply entanglement, which would require a non-factorizable joint quantum state. Demonstration of entanglement would require additional experimental evidence, such as the observation of non-factorizable two-photon correlations, should two-photon emission events be detected at the AIS.

If transient entangled states were shown to occur, they could in principle support the simultaneous evolution of multiple correlated quantum configurations. In quantum information theory, the superposition of quantum states provides the basis for quantum parallelism, whereby a quantum system can evolve along multiple computational pathways simultaneously prior to measurement (Deutsch, 1985; Nielsen and Chuang, 2010). Whether any analogous information-processing advantage could arise from transient entanglement among AIS emitters remains entirely speculative and would require substantial theoretical and experimental investigation.

In this limited sense, the framework bears a conceptual analogy to certain aspects of quantum information systems. However, unlike engineered quantum computers—which require sustained coherence, precisely controlled gate operations, and extensive error correction—the present model proposes only ultrafast and highly transient non-classical correlations occurring on sub-picosecond timescales within a dissipative biological environment. Accordingly, the major challenges associated with maintaining long-lived coherent quantum states in engineered quantum computing systems are not directly applicable here, since the proposed mechanism does not rely on prolonged coherence or fault-tolerant quantum information processing in warm and wet biological tissue.

In the proposed framework, membrane-potential-dependent information is hypothesized to be encoded in the relative phase relationships among ensembles of emitters at the AIS. If such transiently coherent states undergo rapid decoherence, the resulting dynamics may give rise to two conceptually distinct consequences. First, the resulting classical distribution of emitter orientations and phase relationships could reflect local voltage gradients along the proximal-to-distal axis of the AIS, thereby influencing cooperative emission dynamics and, ultimately, neuronal excitability and action-potential generation. Second, any residual non-classical correlations among emitters, if they occur, are proposed to represent information related to certain aspects of a momentary perceptual state.

The first consequence is, in principle, accessible to experimental investigation through measurements of neuronal activity and putative emission signatures. By contrast, the second aspect remains substantially more speculative, as it depends on the existence and functional relevance of non-classical correlations in a biological setting. Establishing a direct relationship between such correlations and perceptual experience would require experimental approaches that are not currently available and therefore remains a long-term hypothesis rather than a demonstrated mechanism.

### 6.1 Non-classical correlation between emitters at the AIS gives way to classical correlation between neuronal activities

Wolf Singer and colleagues proposed that gamma-band oscillations (30–100 Hz) may provide a physiological substrate for temporal binding, enabling distributed neuronal populations to integrate sensory features into unified percepts through millisecond-scale synchrony (Engel et al., 2001; Engel and Singer, 2001). Within this framework, synchronous inputs from distributed neuronal populations may facilitate alignment of neuronal responses during feature integration. In the present model, the proposed physical substrate underlying conscious awareness is instead attributed to transient non-classical electrodynamic correlations among emitters located at the AIS of integrator neurons in the cortex (Figure 3).

Within the proposed framework, during activation of a multipolar cortical neuron, the probability of micro-reversal events is hypothesized to decrease as membrane potential at the AIS approaches the action-potential firing threshold. As the number of micro-reversals approaches zero, a larger fraction of S4 segments may successfully traverse the CTC/HCS without reversal, thereby completing forward activation movement. Consequently, generation of action potential may reflect a state in which cooperative electrodynamic dynamics have shifted toward stabilized forward S4 transitions.

Conversely, failure to initiate an action potential would imply persistence of greater micro-reversal activity, including possible full reversals of S4 movement. In this framework, transient non-classical correlations among emitters are therefore proposed to encode information related to the integrated synaptic input state of the neuron, while neuronal firing represents the macroscopic electrophysiological outcome of those underlying electrodynamic dynamics. Thus, information encoded in transient emitter correlations may also become indirectly represented in the firing behavior of cortical integrator neurons.

More broadly, numerous neurophysiological studies have demonstrated that cognitive processing is associated with correlated neuronal activity across distributed cortical networks. The present framework extends this concept by proposing that transient electrodynamic correlations at the AIS may constitute an additional biophysical layer contributing to neuronal integration underlying unified perceptual awareness as illustrated in Figure 4.

**Figure 4:**
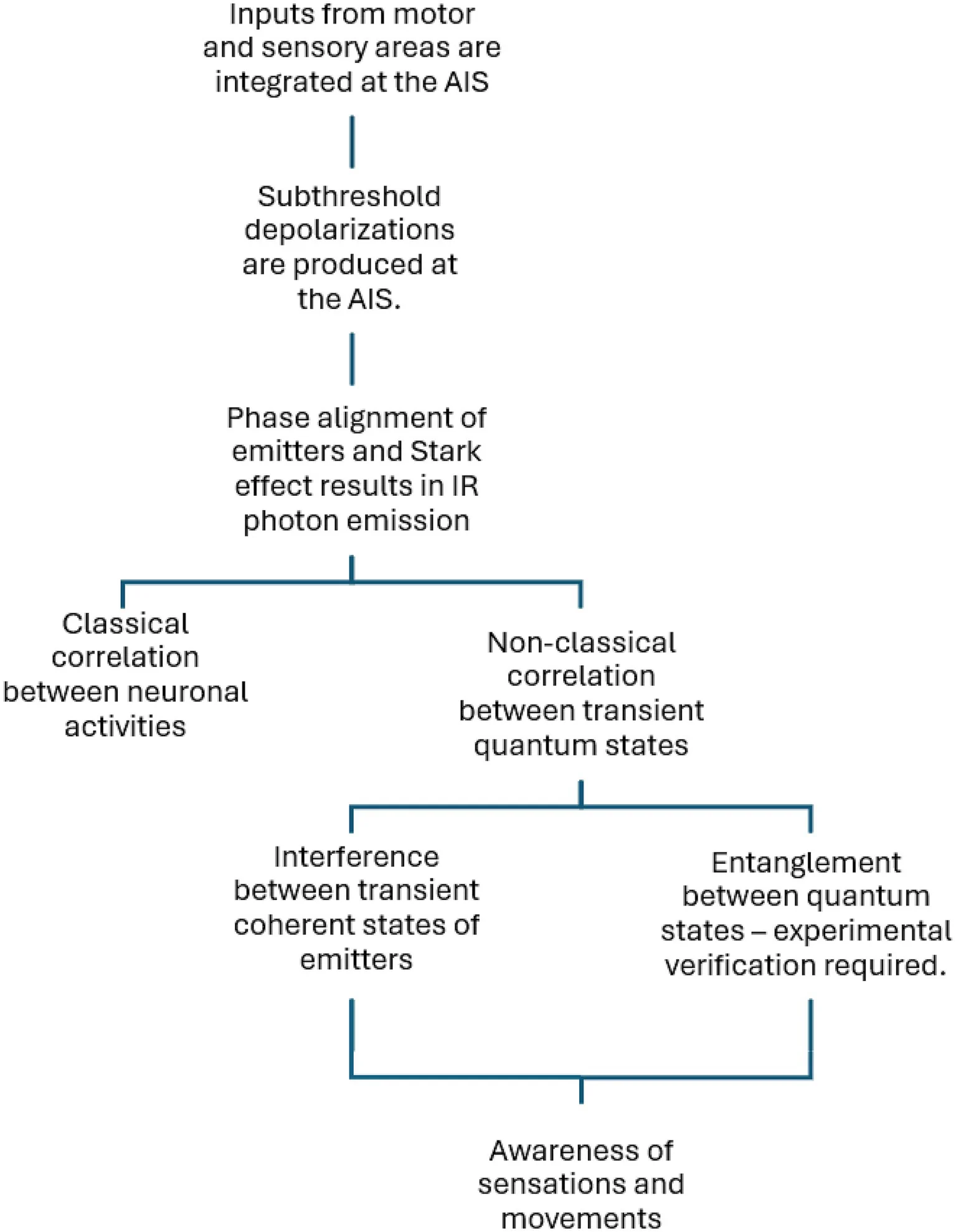
Proposed multiscale framework linking NaV-channel electrodynamic dynamics to neural information processing. Synchronous synaptic inputs from sensory and motor cortical areas converge at the axon initial segment (AIS), where they generate subthreshold membrane depolarization. Within the proposed framework, subthreshold depolarization promotes transient S4 voltage-sensor micro-reversal dynamics, leading to partial phase alignment of arginine-associated emitters and enhanced Stark-effect polarization within the hydrophobic constriction site. These conditions are hypothesized to facilitate transient mid-infrared cooperative emission and short-lived non-classical correlations among emitters. In parallel, conventional neuronal communication gives rise to classical correlations between neuronal activities.

## 7 Experimental predictions

The proposed framework generates a set of specific, testable, and falsifiable predictions that distinguish it from existing classical descriptions of ion channel function. These predictions focus on measurable physical observables—particularly electromagnetic emission and correlation signatures—arising from S4 voltage-sensor dynamics.

### 7.1 Time-locked mid-infrared emission during voltage-sensor activation

We predict that transient mid-infrared (mid-IR) photon emission, if present, would occur in close temporal association with S4 voltage-sensor dynamics. Rather than arising from a single event, emission is expected to occur within multiple, short-lived coherence windows during the rising phase of depolarization, when S4 segments may undergo rapid, small-amplitude micro-reversal oscillations near the charge-transfer center. Each such window may support a brief burst of emission, such that the total photon yield per spike reflects the cumulative contribution of several temporally clustered events. Under voltage-clamp conditions, any emission should therefore be time-locked to gating currents rather than ionic currents, consistent with an origin in electromechanical motion of the voltage sensor rather than ion conduction.

Because mid-IR wavelengths (~3–30 μm) are substantially larger than the diameter of the AIS (~0.5–1 μm), the system operates in a subwavelength regime. Under these conditions, a significant fraction of the electromagnetic field is expected to remain confined near the membrane (near-field). Accordingly, placing a sensitive mid-IR detector (e.g., cryogenic or nanostructure-enhanced InGaAs-based systems adapted for longer wavelengths) within microns of the AIS may improve coupling to this localized field and enhance detection probability.

Although the intrinsic penetration depth of mid-IR radiation in water is on the order of ~10–100 μm, additional absorption, scattering, and geometric confinement in biological tissue are expected to reduce the effective detectable range to the micron scale. Efficient detection is therefore likely to require near-field sensitivity and signal averaging over a large number of coherence-window events.

A critical limitation arises from thermal background radiation. At physiological temperature (300 K), the characteristic thermal energy scale is $\mathrm{kT}\approx 4\times 10^{-21}\;J$ , placing mid-IR photon energies (~10^−20^ J) within a few multiples of thermal energy. As a result, biological tissue and the surrounding environment naturally emit broadband mid-IR radiation, producing a substantial background signal. Consequently, any emission arising from NaV channel dynamics would be extremely weak and detectable only if it exhibits distinguishing features—such as temporal locking to gating currents, nonlinear dependence on membrane potential, or non-classical photon statistics.

Failure to detect emissions above thermal background under these conditions would argue against the proposed mechanism within the sensitivity limits of the experimental setup.

### 7.2 Nonlinear dependence of emission on membrane potential near the activation threshold

Emission intensity is expected to exhibit a nonlinear dependence on membrane potential, with a peak near subthreshold depolarization. This regime corresponds to increased probability of S4 stalling and micro-reversals at the CTC. At more depolarized potentials, where S4 transitions become effectively monotonic, emission should decrease. A monotonic increase in emissions with depolarization would not support this prediction.

### 7.3 Photon statistics indicative of cooperative emission

If multiple emitters contribute collectively, detected photons may exhibit deviations from Poisson statistics, such as photon bunching characterized by a second-order correlation function $g^{\left(2\right)}(0)>1$ . Such signatures, measurable using Hanbury Brown–Twiss interferometry (Dicke, 1954; Gross and Haroche, 1982), would be consistent with cooperative (e.g., Dicke-type) emission. In contrast, $g^{\left(2\right)}(0)\approx 1$ would indicate independent or thermal emission.

### 7.4 Dependence of emission on NaV channel density and AIS organization

Because the hypothesis relies on collective effects, emission should scale with the density and spatial organization of NaV channels. The AIS, which contains a high density of NaV channels arranged in a quasi-periodic lattice, is predicted to exhibit stronger emission and correlation signatures than soma or dendrites. Disruption of AIS actin cytoskeletal organization by cytochalasin D is expected to reduce any cooperative effects.

### 7.5 Spectral characteristics consistent with vibrational modes

Any detected emission is predicted to occur in the mid-infrared spectral range, corresponding to vibrational modes of the guanidinium group of arginine residues within the voltage-sensing domains of NaV channels. Spectral measurements should therefore reveal structured or frequency-specific features rather than broadband thermal radiation. Absence of such spectral signatures would weaken support for the proposed mechanism.

### 7.6 Pharmacological modulation of cooperative emission by NaV emitters at the AIS

Pharmacological or physiological manipulations that alter membrane excitability are expected to modulate any putative emission indirectly by influencing S4 voltage-sensor dynamics. Conditions that stabilize hyperpolarized membrane states—thereby reducing the probability of voltage-sensor activation—are predicted to decrease emission. Conversely, conditions that increase near-threshold activity, where S4 stalling and micro-reversal dynamics are more likely, may enhance emission. These effects would reflect changes in voltage-sensor kinetics rather than direct optical interactions.

Such predictions can be tested using controlled *in vitro* preparations. For example, pharmacological manipulations that stabilize hyperpolarized membrane potentials or reduce the probability of NaV-channel activation could be used to determine whether reduced neuronal excitability is accompanied by diminished emission. Any observed changes should be interpreted in terms of altered gating dynamics rather than downstream physiological or behavioral effects.

A critical experimental control for pharmacological experiments is provided by tetrodotoxin (TTX), a high-affinity blocker of TTX-sensitive NaV channels, including NaV1.6 at the axon initial segment. Application of TTX is expected to suppress voltage-sensor activation and eliminate gating currents under physiological conditions. Accordingly, any emission arising from S4 dynamics should be significantly reduced or absent in the presence of TTX. Persistence of signal despite TTX application would argue against a NaV-dependent mechanism.

### 7.7 Genetic modulation of cooperative emission by NaV emitters at the AIS

Mutations in the NaV1.6-encoding gene SCN8A have been associated with a range of neurodevelopmental phenotypes, including cortical visual impairment. In a cohort study, cortical blindness was reported in 17 of 22 affected individuals (Gardella et al., 2018), all of whom carried heterozygous variants. While this phenotype is generally interpreted as a consequence of severe network dysfunction arising from altered channel activity, it also highlights the sensitivity of cortical processing to perturbations in NaV1.6 function.

Within the framework proposed here, heterozygous mutations introduce biophysical heterogeneity into the population of NaV channels at the AIS. Such heterogeneity could broaden the distribution of transition frequencies, alter gating kinetics, or disrupt local field alignment among emitters, thereby reducing the degree of phase correlation achievable within a coherence window. In this sense, mutations may indirectly modulate the efficiency of any cooperative electrodynamic interactions, including those consistent with Dicke-type emission, although this remains a speculative extension.

This hypothesis can be tested experimentally by comparing mid-infrared emission from axon initial segment–enriched preparations derived from control neurons and those harboring SCN8A variants. A relative reduction or alteration in emission characteristics in mutant preparations—if observed under controlled conditions—would be consistent with the idea that channel-level heterogeneity influences cooperative electromagnetic behavior. However, such findings would need to be interpreted cautiously, given the dominant role of altered excitability and network dysfunction in SCN8A-associated phenotypes.

### 7.8 Modulation of perceptual processing by polarization controlled mid-infrared stimulation

A potentially testable prediction of the present framework involves the application of polarization-controlled mid-infrared (mid-IR) femtosecond pulses to neuronal populations. In this model, cooperative interactions among emitters depend on transient phase coherence and partial alignment of oscillating dipoles within the AIS. Consequently, ultrafast mid-IR fields with varying polarization states or orbital angular momentum (OAM) may perturb these collective dynamics. Fields that alter local phase relationships or dipole orientations could transiently weaken cooperative coupling, whereas those more closely matched to the prevailing electrodynamic configuration may promote or stabilize coherent interactions. Because OAM-carrying photons possess a structured phase profile and can transfer angular momentum to matter (Allen et al., 1992; Yao and Padgett, 2011), they may provide an additional means of influencing collective phase organization beyond that achievable through polarization alone.

This possibility is motivated by the established use of infrared radiation to excite and probe molecular vibrational modes. Ultrafast IR pulses can generate coherent vibrational wave packets and influence conformational dynamics in molecular and protein systems (Mukamel, 1995; Barth, 2007; Hamm and Zanni, 2011). Within the present framework, such stimulation could differentially affect the vibrational dynamics of S4 arginine side chains, which are embedded within the chiral and structurally asymmetric environment of the *α*-helical voltage sensor. Consequently, left- and right-circularly polarized fields may interact differently with local vibrational dipoles and transient electrodynamic states. Any such effects, however, are expected to be weak, short-lived, and strongly constrained by thermal fluctuations and rapid environmental decoherence.

Accordingly, the framework yields a speculative experimental prediction. If polarization-specific or OAM-modulated mid-IR femtosecond pulses delivered near the AIS of neuronal populations involved in face processing were found, under carefully controlled conditions, to transiently alter face-recognition performance, such findings could be interpreted as indirect evidence that cooperative electrodynamic correlations contribute to neural information processing. Within the proposed mechanism, externally applied fields could perturb transient phase relationships among oscillating S4 arginine side chains by modifying dipole orientation, coherence timing, or local electromagnetic coupling among subsets of NaV channels. Because the proposed dynamics are highly localized and short-lived, even modest perturbations of the local electrodynamic environment could potentially influence information integration during perception.

Any interpretation of such findings would require substantial caution. Mid-infrared stimulation is known to affect neuronal activity through established classical mechanisms, including thermal effects, changes in membrane capacitance, and modulation of membrane excitability. Consequently, behavioral or perceptual effects alone would not establish the existence of non-classical correlations or cooperative emission processes. Rather, such observations would motivate further investigation into whether transient cooperative electrodynamic dynamics participate, alongside conventional electrophysiological mechanisms, in neural information integration underlying perceptual awareness.

### 7.9 Temperature dependence and decoherence constraints

Because the proposed mechanism depends on ultrafast, transient vibrational dynamics, emission and any associated correlations are expected to be sensitive to temperature, with higher temperatures accelerating decoherence and reducing detectable coherence-related signatures. A purely thermal origin of emission would instead show different scaling behavior.

### 7.10 System specificity to NaV voltage-sensor dynamics

Systems lacking functional NaV voltage sensors, or containing mutations that alter S4 gating charge movement, are predicted to show reduced or absent emission signatures. Comparative experiments in heterologous expression systems could test whether the effect depends specifically on NaV channel electromechanics.

### 7.11 Spatial confinement of emission

Due to strong absorption of mid-IR radiation in aqueous environments, any emission is expected to be highly localized, occurring on submicron spatial scales near the membrane. Detection of long-range propagating signals would be inconsistent with the proposed mechanism and known optical properties of biological tissue.

### 7.12 Interpretation and scope

It is important to emphasize that detection of any of the above signatures would not, by itself, establish a role in perception or awareness. Rather, these experiments are intended to test the physical plausibility of transient electromagnetic emission and cooperative effects arising from NaV channel dynamics. Establishing a functional link to neural computation or perceptual processes would require additional, multiscale investigation.

It should also be noted that successful detection of any emitted photons would likely require measurements in close proximity to the actin cytoskeleton at the AIS, as the high water content of neuronal tissue is expected to strongly absorb radiation in this spectral range.

## 8 Computational modeling framework for S4–CTC electrodynamic dynamics

The proposed electromechanical interactions between S4 arginine gating charges and the charge-transfer center (CTC), together with their potential electromagnetic consequences, may be investigated using a modular multiscale computational framework designed to evaluate the physical plausibility of the mechanism proposed in this work and to constrain the experimentally testable predictions outlined in Section 7. Importantly, the present study does not claim that existing computational approaches have demonstrated ultrafast micro-reversals, cooperative infrared emission, or non-classical electrodynamic correlations in voltage-gated sodium (NaV) channels. Rather, the approaches described below represent future computational strategies for determining whether the hypothesized dynamics are compatible with established ion-channel biophysics under biologically realistic conditions.

At the molecular level, atomistic molecular dynamics simulations employing enhanced sampling techniques could characterize the conformational dynamics of the S4 voltage sensor during subthreshold activation, including the formation of metastable intermediate states, transient dwell near the CTC, and the energetic barriers governing gating-charge translocation. Such simulations could determine whether localized sub-ångström reversible excursions (“micro-reversals”) of gating charges are physically plausible within the confined hydrophobic constriction site and estimate their characteristic amplitudes, transition pathways, and residence times.

Hybrid quantum mechanics/molecular mechanics (QM/MM) approaches incorporating explicit membrane electric fields could further quantify the magnitude and spatial distribution of local electric fields within the HCS and evaluate their influence on the electronic structure of the arginine guanidinium group. In particular, these calculations could determine whether the local electric field is sufficient to induce measurable polarization of the guanidinium group, modify vibrational transition dipole moments through the vibrational Stark effect, or alter the energetics of transient interactions between gating charges and conserved residues within the CTC (Warshel and Levitt, 1976; Senn and Thiel, 2009). Such calculations would provide quantitative constraints on several experimentally testable predictions, including the predicted dependence of emission on membrane potential and local channel microenvironment.

Electronic structure and vibrational calculations could also be used to determine the normal vibrational modes, transition dipole moments, and infrared spectra of arginine guanidinium groups embedded within simplified models of the HCS. Combined with atomistic simulations, these approaches could evaluate how local protein geometry, hydration, and electric-field strength influence vibrational frequencies, damping rates, and dephasing times within the partially dehydrated CTC environment.

Importantly, the femtosecond-to-picosecond timescales discussed in the present framework are not experimentally measured properties of NaV-channel micro-reversal dynamics. Rather, they represent order-of-magnitude estimates derived from known guanidinium vibrational frequencies, structural constraints of the HCS/CTC region, and experimentally measured ultrafast vibrational dephasing times in proteins (Zheng et al., 2006; Kwak et al., 2007). Future simulations will therefore be required to determine whether the proposed micro-reversal dynamics occur on timescales compatible with these experimentally observed vibrational limits.

At a larger scale, simplified electrodynamic or semiclassical models may combine molecular parameters obtained from these simulations—including dipole moments, charge acceleration, coherence times, and transition dipole moments—to estimate upper bounds for radiated power, photon yield, and the likelihood of cooperative electromagnetic interactions among neighboring NaV channels. Such models cannot establish the existence of cooperative emission but can determine whether radiation from individual emitters is expected to be negligible and whether collective interactions would be required to produce experimentally detectable signals.

Importantly, the present framework does not assume a specific many-body wavefunction or an established quantum state for the NaV-channel ensemble. Rather, computational modeling is intended to determine whether the coupled dynamics of S4 gating charges, surrounding protein residues, membrane electric fields, and local electromagnetic modes can generate transient collective electrodynamic behavior beyond that expected from independent emitters, or whether the observed dynamics are adequately described by classical electromechanical mechanisms alone.

Because a fully integrated simulation spanning electronic, atomic, and electromagnetic length scales is not currently feasible, a hierarchical multiscale modeling strategy is likely to be the most appropriate approach. By progressively constraining individual components of the proposed mechanism—including metastable gating dynamics, local electric-field distributions, guanidinium vibrational properties, and radiative efficiency—future computational studies can define a physically consistent parameter space against which experimental observations may be compared. In this way, computational modeling will serve primarily as a means of refining, constraining, or falsifying the present hypothesis rather than confirming it.

## 9 Discussion

Current neurophysiological frameworks describe neural information processing primarily in terms of action potentials, synaptic transmission, and large-scale network synchrony (Hodgkin and Huxley, 1952; Singer and Gray, 1995; Koch, 1998; Dayan and Abbott, 2001; Buzsáki, 2006; Fries, 2015). These models successfully account for communication and computation within neural circuits; however, the mechanism by which unified perceptual content emerges from distributed neural activity remains incompletely understood. In particular, the feature-binding problem—how distinct sensory attributes such as color, form, motion, and spatial location are integrated into a coherent perceptual experience—continues to represent a major theoretical challenge (Treisman and Gelade, 1980; Singer and Gray, 1995; Engel et al., 2001).

Neuroanatomical and physiological observations suggest that higher-order association cortices may provide an important substrate for such integrative processes, such as feature integration. Supragranular pyramidal neurons within temporo-parietal-occipital association cortex have been strongly implicated in conscious perception and awareness (Koch et al., 2016). These neurons participate in highly interconnected posterior cortical networks capable of integrating distributed sensory and cognitive information. In addition, pyramidal neurons within layers 5 and 6 of fronto-parietal and posterior association cortices receive extensive convergent inputs from multiple sensory modalities, motor-related pathways, and long-range corticocortical projections. As illustrated in Figure 3, this large-scale convergence positions these neurons as integrative hubs that coordinate multimodal information across distributed neural systems (Felleman and Van Essen, 1991; Andersen, 1997; Sherman and Guillery, 2002; Stein and Stanford, 2008; Larkum, 2013; Koch et al., 2016). When synaptic inputs carrying information about different stimulus features converge onto integrator neurons (Figure 3), feature integration is generally understood to arise through established mechanisms of synaptic integration, recurrent cortical processing, and neuronal synchrony. The present framework does not propose an alternative mechanism for feature binding. Rather, it hypothesizes that transient electrodynamic dynamics associated with NaV-channel voltage sensors at the axon initial segment may provide an additional molecular-scale process that could interact with these established network mechanisms. At present, however, it remains unknown whether, or by what mechanism, such stochastic ultrafast molecular dynamics might influence neuronal population activity, oscillatory synchrony, or feature binding. Elucidating these relationships will require future multiscale computational modeling together with experimental studies spanning molecular, cellular, and systems neuroscience.

Additionally, predictive coding theory, a classical computational framework for perception and action, proposes that higher cortical areas generate predictions that are conveyed through feedback pathways, whereas lower areas transmit prediction-error signals through feedforward pathways. These recurrent interactions are distributed across cortical layers and continuously update internal models of the environment (Rao and Ballard, 1999; Friston, 2005; Bastos et al., 2012). Within the present framework, we propose that the convergence of feedforward and feedback activity onto neurons in the deeper cortical layers gives rise to transient cooperative electrodynamic coupling among emitters at the AIS. The resulting collective states are hypothesized to encode information about the integrated neural context and may contribute to the physical basis of conscious awareness.

Within a classical framework, this anatomical organization may provide conditions favorable for temporally coordinated synaptic integration at the AIS, where membrane-potential fluctuations reflect the combined influence of distributed excitatory and inhibitory inputs. As discussed in Section 3.3.3, synchronized activity within cortical minicolumns may also create conditions under which emitters associated with neighboring AISs interact through transient electromagnetic fields, particularly when the participating structures are separated by distances smaller than the wavelength of the proposed infrared emissions. Such interactions, if they occur, could influence the collective dynamics of local neuronal ensembles during periods of synchronized activity.

Under this hypothesis, information processing within cortical minicolumns would remain fundamentally dependent on established mechanisms of synaptic integration and neuronal synchrony. However, transient electrodynamic interactions associated with NaV-channel activity at the AIS may provide an additional ultrafast layer of coordination that complements these classical processes (Figure 4 and Table 4). In this view, functions such as feature integration and sensorimotor integration may emerge primarily from large-scale patterns of neuronal synchronization, while potentially being modulated by localized electrodynamic processes operating on sub-picosecond timescales.

In the present framework, the collective state of emitters is proposed to influence the probability of an action potential initiation, whereas transient correlations among emitter states may encode information related to the synaptic inputs integrated at the AIS. Importantly, the proposed information-processing mechanism relies primarily on transient phase coherence among emitters rather than on quantum entanglement. Voltage-dependent modulation of relative emitter phases may alter patterns of constructive and destructive interference within the collective electromagnetic field, thereby influencing cooperative emission dynamics. Consequently, certain collective emission modes may be enhanced while others are suppressed.

Although entanglement could, in principle, further strengthen collective behavior in idealized quantum systems, it is not required for the cooperative emission phenomena considered here. Transient phase coherence alone is sufficient to support interference-based interactions among emitters. Within the biological environment of the AIS, however, coherence is expected to persist only on sub-picosecond timescales before being disrupted by environmental interactions, implying that any interference effects would be highly localized in space and time. Accordingly, entanglement, if present at all, would represent an additional and presently unverified layer of correlation beyond that necessary for coherent interference.

The potential informational significance of constructive and destructive interference has also been explored in other theoretical frameworks. In earlier work, Stoll (2026) proposed that interference among high-dimensional probability distributions associated with ionic states at neuronal membranes may contribute to information encoding and conscious experience. Within that framework, if neuronal membranes satisfy the requirements of a holographic recording surface, interference patterns are hypothesized to generate holographic representations of encoded information that may be associated with phenomenal content.

In the present proposal, however, there is limited support for a direct role of entanglement in neuronal information processing. Instead, we emphasize the potential role of interference arising from transient phase correlations among emitters during short-lived coherent states. Under such conditions, constructive interference may selectively amplify certain patterns of activity, whereas destructive interference may suppress others. Consequently, interference among emitters could provide a mechanism by which information related to membrane potential fluctuations during synaptic integration becomes differentially encoded within the collective electrodynamic state of the AIS.

A potential experimental test of this framework would be to examine whether externally applied ultrafast mid-infrared pulses, particularly those carrying orbital angular momentum (OAM) or defined polarization states, can perturb transient phase relationships among emitters at the AIS. Any resulting effects on neuronal activity or perceptual processing could provide indirect evidence regarding the functional significance of such transient coherent states.

The spatiotemporal organization of depolarizing synaptic currents converging onto the AIS may generate transient spatial gradients of emitter phases on sub-picosecond timescales, while the membrane potential itself propagates along the AIS on much slower sub-millisecond timescales. Consequently, the instantaneous membrane-voltage gradient along the AIS—which reflects the integration of direct and indirect synaptic inputs associated with environmental interaction—may modulate both emitter dynamics and neuronal firing probability. The resulting firing patterns reduce uncertainty regarding external stimuli within neuronal circuits. According to Landauer’s principle, reduction of informational uncertainty is associated with energy dissipation (Landauer, 1961; Gupta and Bahmer, 2019). Within the present framework, this energy dissipation may provide a physical correlate of perceptual processing, while any transient non-classical phase correlations among emitters could encode information related to the stimulus itself, such as facial features during face recognition.

According to Landauer’s principle, reduction of informational uncertainty is associated with energy dissipation (Landauer, 1961; Gupta and Bahmer, 2019). Within the present framework, this energy dissipation may provide a physical correlate of perceptual processing. Although the present study does not explicitly model the thermodynamic generation and compression of information during neuronal computation, related theoretical work has explored this possibility in cortical networks. Stoll (2024c) proposed that cortical neurons undergo cyclical phases of information generation and compression, whereby noisy fluctuations generate a distribution of possible system states and statistical consistencies within the resulting dataset permit the selection of a single system state. In that framework, information compression is associated with the release of free energy in the infrared spectral range, which may influence neuronal signaling through changes in membrane energetics.

The present work proposes a complementary molecular mechanism that could, in principle, provide a physical substrate through which such infrared-range energy release influences neuronal information processing. During synaptic integration, the AIS integrates convergent inputs from multiple neuronal sources and generates a single action potential that can subsequently propagate along axonal branches to multiple downstream targets. This reduction from multiple synaptic inputs to a single neuronal output may be viewed as a form of information compression. Immediately preceding action-potential initiation, subthreshold membrane depolarization is proposed to promote transient S4 voltage-sensor micro-reversal dynamics within NaV channels, giving rise to short-lived electrodynamic coupling among neighboring emitters.

Within the proposed framework, if information compression is accompanied by spontaneous emission of infrared photons, as predicted earlier by Stoll (2024b), the NaV-channel dynamics described here provide a plausible molecular mechanism by which such infrared energy could arise during subthreshold neuronal processing. Furthermore, the emitted infrared field, if generated cooperatively, could transiently enhance electrodynamic coupling among neighboring emitters through weak field-mediated interactions. Although highly speculative, this sequence would provide a conceptual link between thermodynamic information compression, reduction of uncertainty regarding environmental stimuli, transient cooperative electrodynamic dynamics at the AIS, and subsequent neuronal output. Future theoretical modeling and experimental studies will be required to determine whether such a relationship exists.

We also emphasize that long-distance communication is enabled by classical mechanisms, such as communication through coherence (Fries, 2005, 2015), while perceptual awareness, as speculated here, may result from transient non-classical communication between emitters at the AIS.

Furthermore, any proposed non-classical correlations would remain tightly coupled to the evolving membrane potential and confined to ultrafast temporal windows. Importantly, the framework does not require long-lived quantum coherence or sustained entanglement, but instead proposes transient collective electrodynamic dynamics that may, in principle, provide a bridge between perceptual awareness and macroscopic neuronal activity.

From a thermodynamic computational perspective, neural synchrony may also provide energetic advantages because synchronized activity can compress information and improve predictive efficiency, thereby reducing free-energy expenditure (Stoll, 2024a). Such energetic efficiency may, in turn, promote stabilization or reinforcement of synchronized network states through adaptive modulation of synaptic activity. Within this context, the proposed ultrafast electrodynamic processes at the AIS are not intended to replace established neurophysiological mechanisms, but rather to provide a possible additional layer linking microscopic molecular dynamics to macroscopic neuronal integration and perceptual processing.

The present framework predicts transient mid-infrared photon emission, consistent with the theoretical framework proposed by Stoll (2024b), which likewise predicts endogenous infrared photon generation during cortical information processing. The two frameworks are complementary rather than competing. Stoll’s work provides a thermodynamic and quantum-theoretic formalism linking information processing to infrared photon emission and energy efficiency, whereas the present framework proposes a candidate molecular mechanism in which transient electromechanical dynamics of NaV-channel voltage sensors at the axon initial segment (AIS) could give rise to such emission.

Within the present framework, any cooperative radiative process, including Dicke-type cooperative emission, is hypothesized to depend on transient partial phase alignment among localized subensembles of S4-associated emitters within NaV channels at the AIS. If such phase alignment occurs, it is expected to be extremely short-lived and tightly constrained by the instantaneous electrophysiological state of the neuron. Consequently, sub-picosecond coherence windows would be governed by the spatiotemporal pattern of synaptic inputs converging on the AIS and the resulting membrane-potential dynamics. Under this view, transient phase relationships among neighboring emitters could, in principle, modulate the probability and characteristics of cooperative emission events without requiring long-lived quantum coherence, sustained entanglement, or quantum computation.

Accordingly, ultrafast electrodynamic processes associated with NaV-channel gating could become transiently coupled to neuronal information processing while remaining fully embedded within established classical mechanisms of synaptic integration and action-potential generation. Any proposed quantum-like effects would therefore represent a brief, localized complement to conventional electrophysiological signaling rather than an alternative to it.

Previous critiques of quantum approaches to brain function, most notably by Tegmark (2000), have argued that quantum superposition states at the scale of ions involved in action potentials would decohere on extremely short timescales (~10^−20^–10^−19^ s), far shorter than the duration of neuronal signaling (~10^−3^–10^−1^ s). Importantly, the present framework does not posit that action potentials themselves are quantum objects or that long-lived superposition states underlie neural computation. Instead, we propose that transient, short-lived non-classical correlations may arise locally from electromagnetic coupling among vibrating gating charges within the highly confined, high-field environment of NaV channels.

An additional consideration concerns the strong absorption of mid-infrared radiation by water, which is often cited as a limitation for biologically relevant infrared processes. Importantly, the present framework does not require long-range propagation of mid-infrared photons through aqueous tissue. Rather, any proposed electrodynamic interactions are hypothesized to occur within the highly localized membrane environment of the axon initial segment, where voltage-sensor arginine residues transiently occupy the relatively dehydrated and geometrically constrained hydrophobic constriction site. Although this environment is not expected to eliminate decoherence or optical losses, reduced hydration may modestly decrease coupling to surrounding solvent degrees of freedom compared with fully solvated protein environments. Consequently, transient vibrational correlations occurring on sub-picosecond timescales may remain physically plausible despite the strong mid-infrared absorption properties of bulk water.

This distinction is also relevant in the context of Tegmark’s critique of quantum approaches to brain function (Tegmark, 2000). Tegmark considered long-lived quantum superposition states involving ionic degrees of freedom extending over neuronal dimensions and operating on timescales relevant to action-potential signaling. In contrast, the present framework proposes only highly localized and ultrafast electrodynamic dynamics associated with voltage-sensor activation within a confined protein environment. Accordingly, while rapid environmental interactions remain an important constraint, the assumptions underlying Tegmark’s calculations do not directly apply to the transient sub-picosecond processes considered here.

Importantly, mutations in the NaV1.6-encoding gene SCN8A are associated with impairments across multiple cognitive domains, including global intellectual function, language, executive processing, learning and memory, and visual perception (Larsen et al., 2015; Gardella et al., 2018). Since not all patients exhibited cortical blindness in this study, this variability suggests that additional genetic factors beyond NaV channel alterations may influence the electrodynamic coupling among emitters within the proposed framework. Moreover, these findings are consistent with the broader premise that perturbations in NaV channel function can disrupt integrative neural processes underlying conscious awareness; however, any contribution of non-classical correlations among channel-associated emitters requires further investigation.

The primary contribution of this study is to articulate a set of physically grounded, experimentally accessible predictions that connect established ion channel dynamics to measurable electromagnetic phenomena that may help to bridge the gap between neuronal activity and conscious functions. Accordingly, this framework is not intended to supplant classical models of neural function, which remain robust and well supported, but to examine whether additional ultrafast physical processes might contribute to information integration.

## 10 Future directions

Detection of cooperative or superradiant-like infrared emission from ensembles of Voltage-gated sodium channel at the AIS would primarily indicate the presence of transient electrodynamic correlations among emitters during synchronized subthreshold activation. Within the present framework, such emission would not by itself establish long-lived quantum coherence, quantum computation, or biologically functional entanglement. Rather, it would suggest that closely spaced NaV channels may transiently exhibit weak phase alignment or collective electrodynamic behavior under highly constrained physiological conditions.

Future experimental studies could investigate whether cooperative emission signatures correlate with specific neuronal activation states, membrane-potential fluctuations, or patterns of synchronized synaptic input. If experimentally observed, such correlations may provide insight into how transient electrodynamic interactions coexist with classical electrophysiological signaling at the AIS. In particular, it may be relevant to examine whether cooperative emission events reflect aspects of integrated synaptic activity encoded in local membrane dynamics during near-threshold processing.

Potential non-classical photon correlations could also be explored in future studies if two-photon emission events are experimentally detected. Importantly, the presence of temporal synchrony or shared phase reference alone would not be sufficient to establish photon entanglement. Demonstration of entanglement would require rigorous experimental evidence showing non-classical correlations in photon energy, phase, polarization, or coincidence statistics beyond those expected from classical cooperative emission.

Within the present framework, any hypothetical entangled states would most plausibly arise only during highly transient collective emission events in which multiple emitters behave approximately as a unified radiative ensemble. Even under such conditions, rapid decoherence in the biological environment would be expected to severely constrain both the duration and spatial extent of any non-classical correlations. Accordingly, the present work does not claim that biologically significant entangled photonic states have been demonstrated or are required for neural function.

More broadly, future work should focus first on experimentally accessible questions, including:

- whether endogenous mid-infrared emission associated with NaV channel activity can be detected above thermal background,
- whether such emission depends on membrane voltage or gating-current dynamics,
- whether cooperative photon statistics can be experimentally distinguished from incoherent thermal emission,
- and whether emission properties are altered by pharmacological manipulation of NaV-channel activation or AIS organization.

A major limitation of the present framework is the absence of direct computational modeling of transient Arg-Phe interactions within the hydrophobic constriction site (HCS). The current estimates for micro-reversal amplitudes, coherence intervals, and transient *π*–cation dwell times should therefore be regarded as order-of-magnitude approximations derived from known structural constraints of the HCS/charge-transfer center (CTC) region, protein vibrational timescales, and guanidinium vibrational frequencies reported in the literature, rather than as quantitatively validated parameters.

Future computational studies will be essential to evaluate the physical plausibility of the proposed mechanism. In particular, atomistic molecular-dynamics simulations incorporating experimentally resolved NaV-channel structures, enhanced sampling techniques, QM/MM approaches, and electronic structure calculations could help determine whether the proposed metastable S4 dynamics and transient *π*–cation interactions are energetically feasible and occur on timescales compatible with the estimates presented here. Such studies may also clarify the influence of the intense local electric field within the HCS on guanidinium vibrational frequencies, transition dipole moments, and the stability of transient phase correlations among neighboring emitters. In addition, electrodynamic modeling could establish quantitative bounds on possible radiative efficiencies, coherence intervals, and the strength of collective coupling within ensembles of voltage sensors.

Accordingly, the present work should be viewed as a hypothesis-generating framework that integrates existing findings from NaV-channel biophysics, vibrational spectroscopy, and cooperative emission theory to formulate experimentally and computationally testable predictions, rather than as a definitive mechanistic description of NaV-channel dynamics or neuronal information processing.

Taken together, the proposed framework should therefore be regarded as a speculative, yet experimentally accessible, hypothesis concerning transient electromechanical and electromagnetic processes associated with NaV-channel dynamics at the AIS. Whether such ultrafast processes contribute meaningfully to neural computation, information integration, or perceptual awareness remains unresolved. Addressing these questions will require substantial future investigation, including atomistic simulations, QM/MM calculations, electrodynamic modeling, and direct experimental studies designed to detect and characterize the predicted phenomena.

## 11 Significance statement

Understanding how distributed neural activity gives rise to unified perceptual experience remains a central challenge in neuroscience. Although classical mechanisms—including synaptic integration, action potential generation, and large-scale oscillatory synchrony—account for many aspects of neural computation, they do not fully explain the rapid and selective coordination underlying perceptual binding. Here, we propose a testable, biophysically grounded hypothesis in which transient electromechanical dynamics of NaV channels contribute to this integrative process.

Importantly, the proposed framework does not invoke long-lived quantum coherence or suggest that the brain functions as a quantum computer. Rather, it introduces a transient, mesoscale mechanism through which ultrafast molecular electrodynamic interactions may briefly produce non-classical correlation among populations of NaV channels that may be tested for providing a basis perceptual experience.

This hypothesis generates experimentally accessible predictions, including the potential detection of time-locked mid-infrared photon emission and nonclassical photon statistics associated with voltage-sensor activation. By explicitly linking established NaV channel biophysics to measurable physical observables, the present framework offers a novel and experimentally tractable bridge between molecular-scale dynamics and systems-level questions of neural integration, perceptual binding, and conscious awareness.

## Data Availability

The original contributions presented in the study are included in the article/supplementary material, further inquiries can be directed to the corresponding author.
